# Effect of land use, geology, and seasonality on hydrogeochemical baseline variations in a watershed impacted by human activities in the eastern Amazon

**DOI:** 10.1007/s10661-025-14565-7

**Published:** 2025-09-22

**Authors:** Gabriel Negreiros Salomão, Roberto Dall’Agnol, Gabriel Soares de Almeida, Rafael Tarantino Amarante, Glariston Miranda Mello, Renato Oliveira da Silva Júnior, José Tasso Felix Guimarães, Marcio Sousa da Silva, Danieli Mara Ferreira, Paulo Rógenes Monteiro Pontes, Prafulla Kumar Sahoo, Eduardo Duarte Marques, Emmanoel Vieira da Silva Filho

**Affiliations:** 1 Instituto Tecnológico Vale, Rua Boaventura da Silva, 955, Belém, Pará 66055-090 Brazil; 2 Programa de Pós-Graduação Em Uso Sustentável de Recursos Naturais Em Regiões Tropicais, Instituto Tecnológico Vale, Belém, 66055-090 Brazil; 3 Department of Environmental Science and Technology, School of Environment and Earth Sciences, Central University of Punjab, VPO-Ghudda, Bathinda, 151401 Punjab India; 4 Geological Survey of Brazil (SGB/CPRM), Av. Brasil, 1731, Funcionários, Belo Horizonte, Minas Gerais 30140-002 Brazil; 5 Universidade Federal Fluminense (UFF), Instituto de Química, Outeiro São João Baptista S/N, Centro, Niterói, Rio de Janeiro 24.020-141 Brazil

**Keywords:** Contamination, Potentially toxic elements, Gelado Creek Watershed, Itacaiúnas River, Carajás Mineral Province

## Abstract

Watershed monitoring is vital for ecosystem sustainability and contamination source identification. Such studies are scarce in the Amazon region. To address this, an integrated study was conducted to assess variations in baseline concentrations of Fe and potentially toxic elements in the Gelado Creek Watershed (GCW), eastern Amazon. This watershed, situated in the Carajás Mineral Province, hosts two world-class Fe mines with tailing dams. It has also faced extensive deforestation due to rapid human settlement and artisanal mining. Surface water samples were collected at 12 sites within the GCW during 25 monthly field campaigns from April 2016 to April 2018, and physicochemical analyses were conducted. Data processing included statistical treatment, geospatial characterization, multivariate analysis, and hydrogeochemical baseline determination. Time-series maps and multivariate hydrogeochemical patterns revealed four main catchment groups. One group, influenced by a preserved forested area, was considered a reference for determining the hydrogeochemical background. The other three were influenced by pasturelands, urban areas, and mining tailing dams. The highest concentrations of Fe, Ag, Ba, Cd, and Hg were observed at the site influenced by an urban area. High concentrations were also observed in areas with predominant pastureland due to soil exposure and runoff. Moderate to high dissolved Mn concentrations were registered, potentially from water-sediment-tailings interactions and high precipitation events. This hypothesis requires validation through robust chemical analysis and detailed assessment of extreme precipitation. This integrated assessment is a powerful tool for identifying contamination sources, highlighting the need for similar studies to understand geogenic and anthropogenic impacts in the Amazon.

**Supplementary Information:**

The online version contains supplementary material available at 10.1007/s10661-025-14565-7.

## Introduction

Watershed-based monitoring (WBM) of water quality parameters is necessary to ensure that water downstream meets water quality standards. The establishment of a water quality monitoring network provides an effective means to assist the identification of sources of alteration. It is particularly relevant in highly impacted areas by anthropogenic activities, which include agricultural residues (Simedo et al., 2018), urban sewage (Li et al., 2019), artisanal mining (Salomão et al., 2023), industrial effluent (Wang & Yang, 2016), and atmospheric particular deposition (Mamun et al., 2023). The implementation of WBM can provide a vast amount of physicochemical and biological data, collected in a given frequency (e.g., hourly, monthly, or once in each season), which depends on the scale (e.g., local, regional, or continental) of the area of interest. These data enable in-depth studies about the quality of the streams in terms of contaminants, which could lead to a better understanding of the study area and strongly support a more consistent evaluation of regional background and baseline variations in trace element concentrations.

The concepts of background and baseline are still under debate and are sometimes wrongly used as a synonym (Matschullat et al., 2000; Meloni et al., 2023; Reimann & Garrett, 2005; Reimann et al., 2005). In this study, the geochemical background is considered to be a naturally occurring concentration range of an element or chemical compound (Reimann & Caritat, 2005), whereas the geochemical baseline is the present concentration of a given chemical substance in a contemporary environmental sample (Darnley et al., 1995; Gałuszka & Migaszewski, 2012). Both concepts can be used to define reference values, which can be used as a datum to monitor changes over time. Johnson and Demetriades (2011) highlight the dependence of the sampling protocol and analytical methods on a rigorous estimation of geochemical background and baseline concentration values. In this study, we also use the terms hydrogeochemical background and hydrogeochemical baseline due to the nature of the data used in this research, which focuses on surface waters. It is important to acknowledge that groundwater was not considered in this study. Groundwater is connected to surface water and can significantly influence its chemistry. This represents a limitation, and future investigations should incorporate groundwater data to provide a more comprehensive understanding of hydrogeochemical processes.

In terms of calculation, Gałuszka (2007) suggests three different approaches of determination: direct (geochemical), indirect (statistical), and integrated. A wide range of robust statistical methods used to derive geochemical background concentration values is described in Matschullat et al. (2000) and Reimann et al. (2005). Previous studies have focused on establishing geochemical background values considering different approaches at variable scales, which include (i) regional assessment (RA), focusing on deriving background values for a given area, considering the period of sampling, at national (Johnson, 2005; Lister et al., 2005; Reimann & Caritat, 2017; Salminen et al., 2005; Smith et al., 2014; Xie et al., 1997) and regional scales (Sahoo et al., 2019, 2020; Salomão et al., 2020) and (ii) site-specific assessment (SSA), dedicated to evaluate changes over time at local scale, particularly for pristine areas (Correa-Burrows et al., 2021; Gassama et al., 2021; Gustavsson et al., 2012; Levitan et al., 2014; Sequeira et al., 2020).

**Fig. 1:**
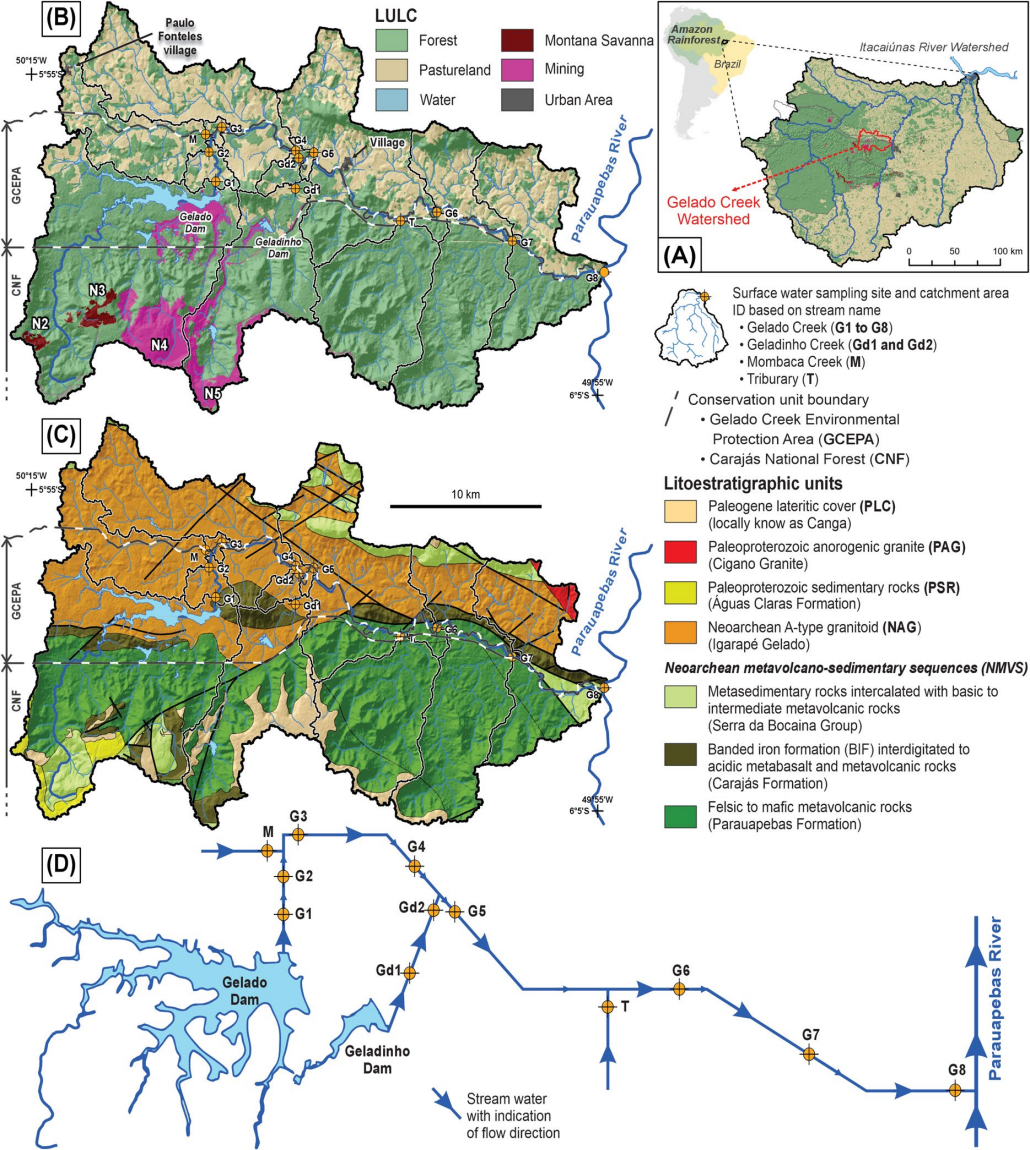
Location of the Itacaiúnas River Watershed (IRW) in the eastern Amazon rainforest (**A**), land use and land cover (LULC) of the year 2017 (**B**), and geological (**C**) maps of the Gelado Creek Watershed (GCW). Schematic diagram of stream flow (**D**) with the indication of the 12 sampling sites located in the GCW; the arrow indicates the flow direction

In Brazil, geochemical mapping projects applied to mineral exploration have been extensively conducted by the Geological Survey of Brazil (GSB, formally known as Companhia de Pesquisa de Recursos Minerais - CPRM), focusing mainly on relevant mineral provinces of Brazil, which include Carajás in the Amazon and Quadrilátero Ferrífero in the Minas Gerais state. However, geochemical mapping projects applied to environmental studies are still scarce, especially in the Amazon rainforest region, North of Brazil. The Vale Institute of Technology (ITV) conducted a large geochemical mapping and background project, namely ItacGMBP, covering the entire Itacaiúnas River Watershed (IRW; Fig. 1A) in the Carajás Mineral Province. The results presented in that project (Sahoo et al., 2019, 2020; Salomão et al., 2020, 2021) revealed (i) the Fe spatial distribution and anomalies and potentially toxic elements (PTE; Ag, As, B, Ba, Bi, Cd, Co, Cr, Cu, Hg, Mn, Mo, Ni, Pb, Sb, Se, Sn, U, V, and Zn). The PTE are dominantly controlled by the geological setting of the area, rather than anthropogenic influences; (ii) the Carajás Basin (CB) domain, one of the four geochemical domains of the IRW, is naturally enriched in several PTE and was classified as a multielement geochemical province, in which the concept of mineralized background values was applied. Besides these two extremely relevant outcomes, it is important to highlight that this project was conducted on a regional scale of observation and sampling, which, depending on the degree of impact, may not precisely guarantee the identification of anthropogenic sources of contamination. The possibility that local anthropogenic impacts occurred in the Itacaiúnas watershed but went undetected cannot be ruled out.

The Gelado Creek Watershed (GCW) is a highly relevant area of investigation for several reasons, which include (i) it is located in the CB domain, a naturally enriched area for several elements (e.g., Fe, Cu, and Mn), due to the occurrences of specific lithologies and mineralizations in the region; (ii) the GCW encompasses important mineral deposits (N2 and N3) and two large open pit Fe mines (N4 and N5), the latter with two tailing dams (Gelado and Geladinho dams) (Fig. 1B); and (iii) the watershed exhibits a strong contrast of land use and land cover (LULC), where the preserved tropical rainforest coexists with areas impacted by human activities, dominated by deforestation for cattle grazing and small-scale farming (Fig. 1A and B). This contrast is intensified by the presence of mining operations and several smaller rural communities with limited infrastructure. As a result of these combined factors, there is a high probability of environmental impacts associated with the interaction of mining activities, agricultural expansion, and dispersed human occupation within the watershed.

This study aims to provide an integrated evaluation of surface water geochemical data from monthly monitoring sites to assess the influence of environmental and geospatial factors on surface water geochemistry and to estimate baseline concentrations in a watershed impacted by anthropogenic activities in the eastern Amazon. The study was designed to answer the following questions:

- Is there any evidence of anthropogenic impacts in the area?
- What are the effects of the seasonality drivers, LULC, and local geological setting on the variation of Fe and PTE baseline concentrations in the GCW?
- What is the relevance of these factors to the definition of local environmental guidelines in surface water?

## Materials and methods

### Study area and geological setting

The study area is the GCW, which covers an area of 543.6 km^2^ and represents an important sub-basin in the context of the Itacaiúnas River Watershed hydrological system in the southeastern Amazon (Fig. 1A). The region is characterized by a tropical monsoon climate (Am), with a mean annual air temperature of 27.2 °C and an average relative humidity of approximately 80% (Silva et al., 2021). It presents two well-defined seasons: a rainy season, from November to May, with an average monthly rainfall of 1550 mm, and a dry season, from June to October, when the average monthly rainfall drops to 350 mm.

The sampling sites are plotted over the LULC (Fig. 1B) and geological layers (Fig. 1C). In terms of LULC types, the GCW can be divided into two separate areas, which are closely related to the boundaries of environmental protection areas:

- **The northern portion of GCW** includes the majority of pasturelands in the region, including the Igarapé Gelado Environmental Protection Area (IGEPA; in Portuguese, Área de Proteção Ambiental do Igarapé Gelado), with the same name as the creek under investigation in the present study. Human occupation here is predominantly rural, with land use focused on cattle ranching and small-scale agriculture (Théry et al., 2019). The main urban area is Paulo Fonteles Village, located in the northwestern part of the GCW near the Mombaca Creek headwaters (see sampling site M in Fig. 1B). The village has an estimated population of around 3000 inhabitants, who are primarily engaged in agricultural, livestock, and local commercial activities (Théry et al., 2019). In addition, there are several smaller unnamed settlements and scattered rural households located along the Gelado Creek, which also contribute to local anthropogenic pressures. These communities generally lack basic sanitation infrastructure, organized waste management, and sewage treatment, which directly influences the hydrochemical quality of surrounding water bodies.
- **The southern portion of GCW** which includes the Carajás National Forest (CNF; in Portuguese, Floresta Nacional de Carajás) corresponds to a preserved area of the Amazon Forest and its coexistence with important world-class active open pit Fe mines (N4 and N5; Fig. 1B). Two dams related to mining activities are the Gelado (G) and Geladinho (Gd) (Fig. 1B). In these dams, the fine-grained tailings resulting from iron ore milling and concentration have been accumulated over the last decades. In the Geladinho Dam, tailings were exploited for reuse in 2010. In the Gelado Dam, the exploitation of tailings started in March 2023. Areas of Montana savanna occur within this area, mainly represented by the N2 and N3 plateaus, which are important targets for Fe exploration in the future.

The significant differences in terms of legislation and human occupation between the IGEPA and areas of the CNF can be attributed to their respective histories and conservation goals, primarily within the GCW. Although it is a protected area, the IGEPA has a long history of human occupation, dating back to May 1989. This has resulted in intense environmental degradation, mainly through the conversion of forests into pastures. The continuous occupation and authorization for various activities are characteristic of the IGEPA. In contrast, the CNF, despite including areas of mining (N4 and N5; Fig. 1B) for many years, the majority forested area preserved. This is due to the emphasis on biodiversity conservation and stricter regulations on exploitation activities in national forests, which has resulted in the preservation of forests in a large part of the IRW. This divergence in conservation policies and human occupation is crucial to understanding the disparities between the two conservation units within the GCW.

The GCW is located in the Carajás Province, an Archean terrain comprising subordinate Proterozoic units (Machado et al., 1991). This Province is divided into four geological domains (Dall’Agnol et al., 2013, 2017): Rio Maria, Sapucaia, Canaã dos Carajás, and Carajás Basin. The study area is located entirely in the Carajás Basin. The geology of the GCW can be simplified into five groups as follows:

- **NMVS:** Neoarchean mafic to felsic metavolcano-sedimentary rocks of the Itacaiúnas Supergroup (Docegeo 1988; Gibbs et al., 1986; Vasquez et al., 2008), represented in the area by the Parauapebas and Carajás formations, both from the Grão Pará Group and by the Serra da Bocaina Group (Costa et al., 2016). The Carajás Formation is composed mainly of banded iron formation, which holds the world-class Fe deposits of Carajás (Gibbs et al., 1986; Martins et al., 2017). These geological units dominate in the southern part of GCW (Fig. 1C).
- **NAG:** Neoarchean A-type like granitoid, represented by the Igarapé Gelado granitic batholith, composed dominantly of highly deformed monzogranites to alkali-feldspar granites (Barros et al., 2009), which occupy most of the northern GCW (Fig. 1C).
- **PSR:** Paleoproterozoic sedimentary rocks of the Águas Claras Formation (Nogueira et al., 1995), partially superposed the Archean units (Fig. 1C).
- **PAG:** Paleoproterozoic anorogenic granites, represented by the Cigano Granite (Dall’Agnol et al., 2005; Machado et al., 1991; Teixeira et al., 2019), restricted to the eastern part of the GCW (Fig. 1C).
- **PLC:** Paleogene lateritic cover, locally known as Canga, composed of Al–Fe duricrust derived from NMVS and associated with hypabyssal mafic rocks (Costa, 1997; Horbe & Costa, 2005).

### Experimental design and analytical methods

The monitoring sites were chosen based on the catchment topography, the direction of main streams, and for evaluating the spatiotemporal variations in surface water quality related to anthropogenic influences (Mello et al., 2021). Previous evidence (Sahoo et al., 2019) indicates that the northern GCW was more exposed to contamination than its southern area, which is covered by preserved tropical forest (Carajás National Forest; Fig. 1B). For this reason, the selected sites for water quality monitoring are concentrated in the northern area. Figure 1D shows a simplified diagram that synthesizes the water flow system in the GCW towards the Parauapebas River and the location of the selected sampling sites presented in Fig. 1. This study considered 12 sampling sites of surface water (G1 to G8 situated in Gelado Creek; Gd1 and Gd2 in Geladinho Creek; M in Mombaca Creek; and T in a tributary). Twenty-five field campaigns were conducted monthly from April 2016 to April 2018 for sampling all monitoring sites (Fig. 1), generally following a regular interval in the first 2 weeks of each month to ensure temporal consistency. Sampling was not performed at the G4 site in June 2017 and at the G6 site in February and March 2018 due to field limitations and access. Samples were classified according to the seasonality of the region, rainy season from November to May, and dry season from June to October.

All samples were collected, prepared, and analyzed by personnel from certified and accredited laboratories (SGS Geosol in Parauapebas-PA and Vespasiano-MG), which followed strict protocols for sampling and analysis, primarily based on the 23rd edition of the Standard Methods for Examination of Water and Wastewater - SMEWW (American Public Health Association - APHA 2017). Two water samples were collected at each site: one unfiltered sample for total metal concentration analysis and one filtered sample (0.45 µm) for dissolved metal analysis. Filtration was carried out on-site using pre-cleaned syringes and disposable membrane filters to minimize contamination. In situ measurements of temperature, pH, dissolved oxygen, electrical conductivity, and turbidity were also recorded using calibrated portable multiparameter probes. Only those parameters relevant to the present study were selected for interpretation. Table 1 summarizes information regarding the analyzed parameters, analytical reference and instrumentation, the detection limit (DL), the unit of detection, and the number of samples below DL based on the seasonality of the region.

**Table 1:** Water quality parameters were analyzed in samples of 12 monitoring sites of the Gelado Creek Watershed during 25 field campaigns conducted monthly (April 2016 to April 2018)

Parameters	Analytical reference*	Method/instrumentation	Detection limit (DL)	Unit	Rainy season		Dry season	
					***n***	**% < DL**	***n***	**% < DL**
Dissolved oxygen	4500 O, G	In situ measurement with multiparameter probe	2	mg/L	174	0	119	0
pH	4500 H^+^ B		2 to 13	-	178	0	119	0
Temperature	2550 B		-	°C	177	0	119	0
Electrical conductivity	2510 B		2	µS/cm	177	0	119	0
Turbidity	2130 B		0.3	NTU	177	0	119	0
Total dissolved solids	2540 C	TDS dried at 180ºC	11	mg/L	177	0.6	119	0
Total suspended solids	2540 D	TSS dried at 103–105 °C	11		105	40.7	119	83.2
Biochemical oxygen demand	5210 B	5-day BOD test	3		177	100.0	119	100.0
Chemical oxygen demand	5220 D	Open reflux method	26		177	84.2	118	94.1
Hardness	2340 B	EDTA titrimetric method	2		166	0	119	0
Total alkalinity	2320 B	Titration method	6		177	1.1	119	0.8
P _total_	4500 P, B, E	Ascorbic acid method	0.02		178	77.0	119	84.0
Ammonia nitrogen	4500-NH_3_ B C	Titrimetric method	0.06		178	55.1	119	73.9
Nitrate	4110 B	Ion chromatography	0.02	mg/L	177	9.6	119	5.0
Nitrite			0.02		178	86.0	119	91.6
Sulfate			1		177	14.7	119	16.8
Sulfide			0.002		177	97.2	119	100.0
Fluoride			0.05		177	85.9	119	91.6
Chloride			1		177	1.1	119	1.7
Al_Dissolved_	3120 B	ICP-AES	0.05	mg/L	177	54.8	119	35.3
Cu_Dissolved_			0.009		178	98.3	119	100.0
Fe_Dissolved_			0.1		177	15.3	119	11.8
Mn_Dissolved_			0.025		177	5.6	119	9.2
Ag_Total_	3120 B	ICP-AES	0.005	mg/L	177	99.4	119	99.2
B_Total_			0.2		177	100.0	119	100.0
Ba_Total_			0.01		177	0.6	119	0.8
Be_Total_			0.004		177	97.2	119	100.0
Cd_Total_			0.001		177	98.3	119	100.0
Co_Total_			0.01		177	99.4	119	94.1
Cr_Total_			0.01		178	90.4	119	95.0
Cu_Total_			0.009		177	93.2	119	100.0
Fe_Total_			0.1		177	0.6	119	1.7
Li_Total_			0.1		177	100.0	119	100.0
Mn_Total_			0.025		177	2.8	119	6.7
Ni_Total_			0.01		178	98.3	119	98.3
Pb_Total_			0.01		178	99.4	119	100.0
V_Total_			0.02		177	100.0	119	100.0
Zn_Total_			0.1		178	98.9	119	100.0
As_Total_	3125 B	ICP-MS	0.004	mg/L	178	99.4	119	100.0
Hg_Total_			0.0002		178	93.3	119	96.6
Sb_Total_			0.005		177	98.3	119	100.0
Se_Total_			0.01		178	100.0	119	100.0

*APHA (2017); *n* number of samples; %<*DL* percentage of data <DL; *ICP-AES* Inductively Coupled Plasma - Atomic Emission Spectrometry; *ICP-MS* inductively coupled plasma mass spectrometry

### Geochemical and geospatial data processing and exploratory analysis

**Fig. 2:**
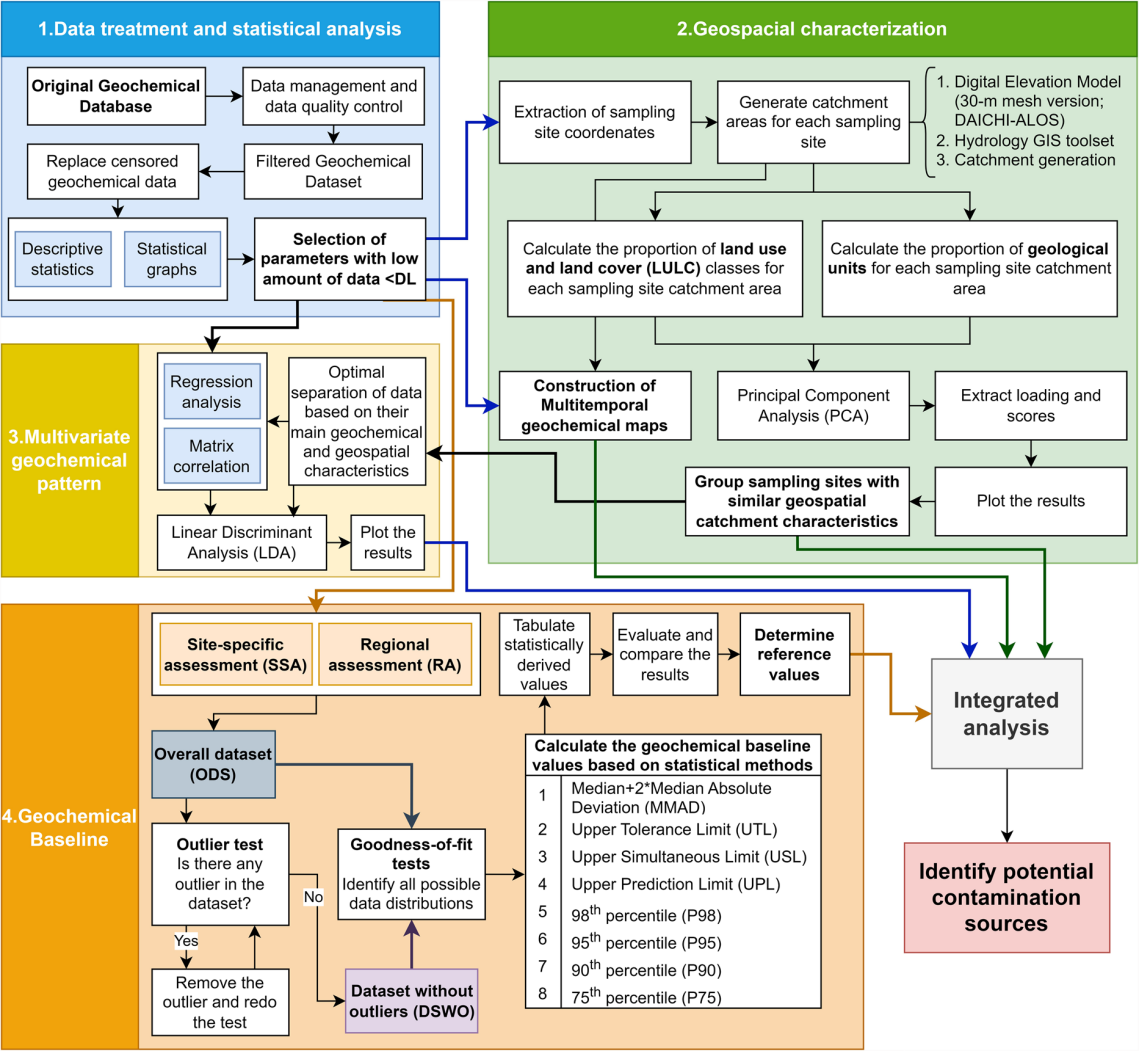
Summary of the integrated geochemical and geospatial data processing and exploratory analysis applied in this study

The general data processing applied in this study is summarized in four main stages (Fig. 2): (i) data treatment and statistical analysis; (ii) geospatial characterization of the study area using geoprocessing techniques to evaluate the areal proportion of LULC and geology within the catchment area of each sampling site; (iii) multivariate geochemical pattern to verify similarities and differences among each sampling site water chemistry; and (iv) hydrogeochemical baseline determination based on modern statistical applications in an integrated assessment. Data analysis was performed using the R programming language in RStudio (RStudio Team, 2020), which is associated with a combination of R packages (Lorenz & Diekoff, 2017; R Core Team, 2022; Venables & Ripley, 2002; Wickham, 2016). Maps and geoprocessing tools were processed in ArcGIS PRO software (Esri, 2023). Geochemical baseline values were determined in RStudio and ProUCL software (United States Environmental Protection Agency - USEPA, 2022). See Appendix A for a detailed description of each stage.

## Results and discussion

### Site catchment analysis integrating LULC and surface geological data

The percentage of the LULC (Fig. 1B) and geological surface layers (Fig. 1C) of each sampling site is presented in Table A.1. These results were submitted to a principal component analysis (PCA) to reduce dimensionality and to evaluate similarities among the sampling site catchments in terms of the main geospatial factors. As a result, Fig. 5A shows the PCA biplot (PC1 vs. PC2), which clearly groups the 12 sampling site into four distinct geospatial affinity classes: (i) natural (NAT): represented only by the sampling site T; (ii) surface water influenced by mining tailings (SWMT): represented by the sites G1, G2, Gd1, and Gd2; (iii) pastureland (PL): represented by the sites G3 to G8; (iv) urban area associated with pastureland (UA): represented only by the sampling site M.

Figure 5A shows that the geospatial classes NAT and SWMT have similar characteristics in terms of LULC, mainly represented by dense natural vegetation coverage (> 72%) and the dominance of PLC and NMVS lithologies (Fig. 1C). However, NAT has a native natural vegetation cover, while SWMT is influenced by water from Fe-tailing dams. PL is clearly defined by the remaining sampling points located at Gelado Creek, which are directly influenced by NAG rock type (Fig. 1C), pasturelands, and other anthropic uses in the area. UA is mainly influenced by NAG but differs clearly from the remaining groups due to the significant contribution of the urban area (Table A.1), which is also related to pastureland in the catchment (see Paulo Fonteles village in Fig. 1B).

### Surface water hydrogeochemical characteristics and multivariate patterns

This study investigated 42 water quality parameters (Table 1). The proportion of < DL values is generally greater during the dry season than in the rainy season, except for nitrate, Al _Dissolved_, Fe _Dissolved_, and Co _Total_. The proportion of analytical values < DL revealed (i) 17 parameters presented represent more than 98% of the data < DL, in at least one season; (ii) chemical oxygen demand (COD), nitrite (NO_2_^−^), fluoride (F^−^), Cr _Total_, and Hg _Total_ account for between 85 and 97% < DL; (iii) total suspended solids (TSS), ammonia nitrogen, P _Total_, and Al _Dissolved_ presented 35 to 85% of data < DL; and, (iv) only 16 water quality parameters (total dissolved solids (TDS), dissolved oxygen (DO), pH, temperature, electrical conductivity (EC), turbidity, hardness, total alkalinity, sulfate (SO_4_^2−^), nitrate (NO_3_^−^), chloride (Cl^−^), Fe _Total_, Fe _Dissolved_, Mn _Total_, Mn _Dissolved_, and Ba _Total_) were found in a substantial amount of analytical data, reasonably acceptable for further processing using multivariate statistics methods. Heatmaps were constructed for selected PTE (Fig. 4) and time-series distribution maps for Fe _Total_ (Fig. 5), Fe _Dissolved_, Mn _Total_, Mn _Dissolved_, Al _Dissolved_, and Ba _Total_ (Appendix B) to identify spatial patterns and anomalous concentrations above the regional reference level.

**Fig. 3:**
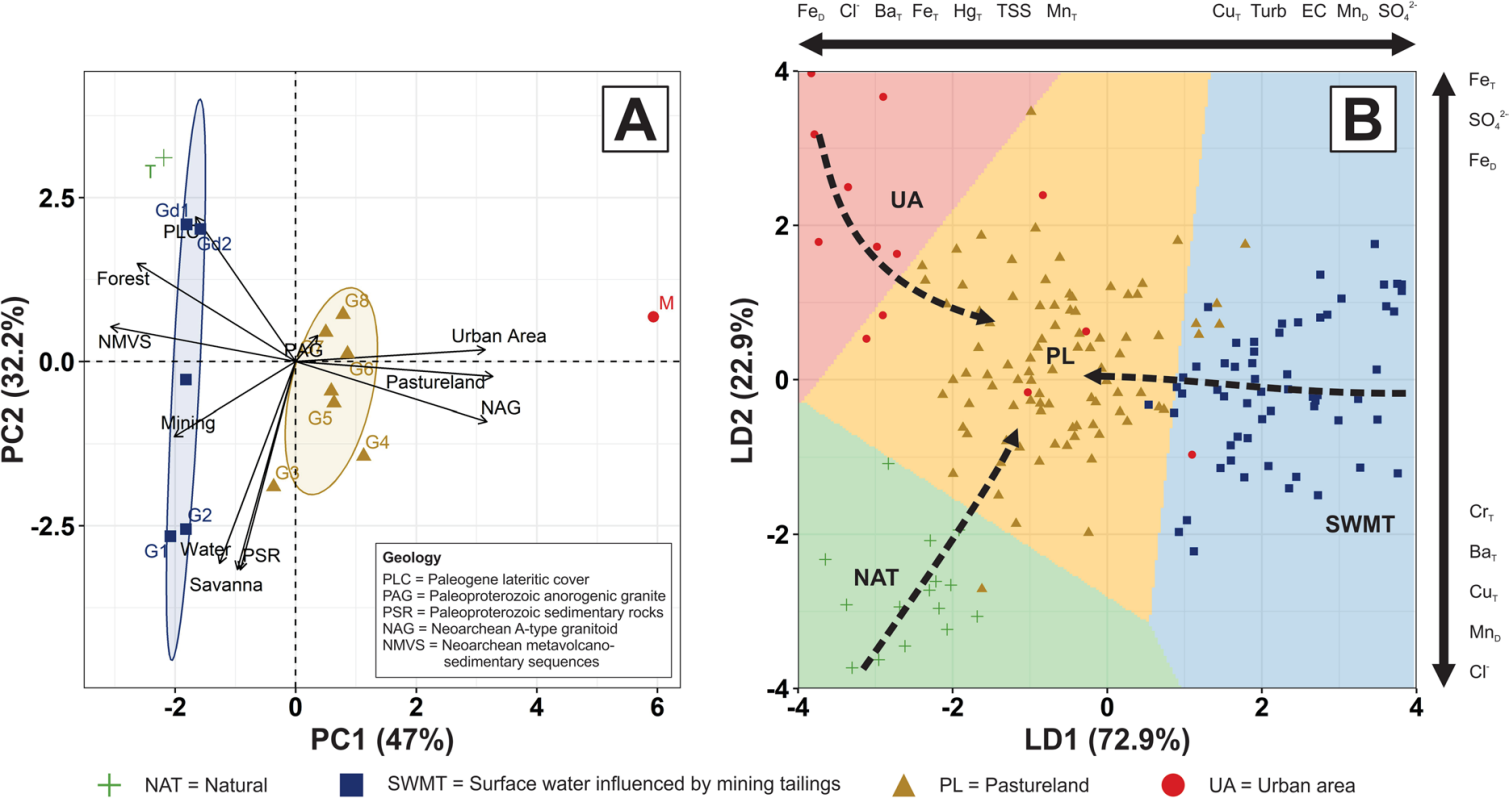
(**A**) Principal component analysis biplot (PC1 vs. PC2) of the percentage share of each class of land use and land cover (LULC) and geology (see Table A.1) calculated for the 12-sampling site catchment area. (**B**) Linear discriminant analysis of selected water quality parameters considering the geospatial classification presented in Figure 6A

The geochemical data was submitted to a linear discriminant analysis (LDA) to evaluate a combination of water quality parameters that characterize or separate the four geospatial classes described in the site catchment analysis (Fig. 3A). At first, all parameters were considered for investigation. However, in order to improve the LDA model accuracy, only the most relevant water quality parameters were considered in the final model, which includes Fe _Dissolved_, Fe _Total_, Mn _Dissolved_, Mn _Total_, Al _Dissolved_, Ba _Total_, Hg _Total_, Cu _Total_, Cr _Total_, TSS, SO_4_^2−^, NO_3_^−^, Cl _Total_, turbidity, and EC. The selection of these parameters was based on their relative weight and contribution to the explained variance; parameters with minimal influence on class separation were removed, keeping only those that significantly enhanced the discrimination among groups. The LDA result is presented in Fig. 3B (model accuracy = 86.4%). The discriminant functions are given below for LD1 (1) and LD2 (2), and the proportion of explained variance is 72.9% and 22.9%, respectively. Only the most relevant parameters were displayed in the equations. For the entire discriminant function, consider Table A.2.

1 $$\begin{aligned}\text{LD}1&=1.212{[\text{SO}}_{4}^{2-}]+0.618\left[{\text{Mn}}_{\text{D}}\right]+0.241\text{EC}+0.216\text{Turb}+0.207\left[{\text{Cu}}_{\text{T}}\right]+\dots -0.255[{\text{Mn}}_{\text{T}}]\\&-0.297\text{TSS}-0.323[{\text{Hg}}_{\text{T}}]-0.347[{\text{Fe}}_{\text{T}}]-0.357[{\text{Ba}}_{\text{T}}]-0.451[{\text{Cl}}^{-}]-0.457{[\text{Fe}}_{\text{D}}]\end{aligned}$$

2 $$\begin{aligned}\text{LD}2&=0.998\left[{\text{Fe}}_{\text{T}}\right]+0.783{[\text{SO}}_{4}^{2-}]+0.743\left[{\text{Fe}}_{\text{D}}\right]+\dots -0.206\left[{\text{Cr}}_{\text{T}}\right]\\&-0.276\left[{\text{Ba}}_{\text{T}}\right]-0.330{[\text{Cu}}_{\text{T}}]-0.559{[\text{Mn}}_{\text{D}}]-0.86[{\text{Cl}}^{-}]\end{aligned}$$

Similar methodological approaches were conducted in previous studies, demonstrating high standard achievement in source identification and characterizing groups of samples by using hydrogeochemical data (Bi et al., 2021; Braun et al., 2013; Xue et al., 2023). In this study, the integrated assessment (PCA for the site catchment analysis and LDA for the hydrogeochemical data) results demonstrate that, in general, the surface water geochemistry of each of the four groups is significantly different, highlighting natural influences and anthropogenic impacts in the GCW, which are discussed in the following sections.

#### Hydrogeochemical signature of natural surface water

The T sampling site is located at the outlet of a catchment entirely situated in a preserved area covered by forest, where human impact can be considered insignificant (Fig. 1 and Table A.1). NAT class area is plotted on the left lower corner of the LDA biplot diagram (Fig. 5B), indicating influence from the group of parameters with negative scores of both, LD1 and LD2. Chloride and Ba _Total_ are important contributors to the geochemical characteristics of natural surface water samples collected at the T site, as they influence both discriminant functions.

Chloride concentrations at the T site range from 2.01 to 4.26 mg L^−1^, which is a relatively low concentration range when compared with marine environments or those impacted by anthropogenic activities. Chloride in surface water can originate from various sources, including atmospheric deposition (mainly during the rainy season) and catchment rocks (Sahoo et al., 2019). According to De Vos (2006) , in natural areas where the precipitation does not entirely explain the chloride concentrations, the mineralogy of the area may play a significant role. For instance, apatite [Ca_5_(PO_4_)_3_(OH, F, Cl)] can be a Cl-bearing mineral; it is an important accessory mineral of granitoids and mafic metavolcanic rocks (NMVS) of the Carajás Basin and also of the iron-oxide copper gold (IOCG) mineralization system of the northern copper belt of Carajás (Diniz et al., 2023; Moreto et al., 2014; Trunfull et al., 2020), which occurs in the GCW. The geological setting of the area supports with the understanding of Cl concentrations associated with unusually high concentrations of Cu (total and dissolved) detected at the T site (Fig. 3). In addition, chlorine can replace hydroxide ions in biotite [(K(Mg, Fe)_3_(Al, Fe)_1_(Si_3_Al)O_10_(OH)^2^] and amphiboles [(Ca, Na)_2–3_(Mg, Fe^2+^, Al)_5_(Al, Si)_8_O_22_(OH)_2_] (De Vos et al., 2006), which may be an additional source of Cl. This hypothesis is reinforced by the fact that amphiboles and biotites of Neoarchean granites of Carajás Province, like the Igarapé Gelado granite, are relatively enriched in chlorine (Dall’Agnol et al., 2017).

The distribution of Ba _Total_ in surface water of the IRW (Fig. 1A) was presented in a previous study (Sahoo et al., 2019), revealing high concentrations in the north of the area, limited to the Bacajá Domain ($\overline{x }$ = 0.08 mg L^−1^; max = 0.4 mg L^−1^), and in the south towards the Mesoarchean granitic domains ($\overline{x }$ = 0.12 mg L^−1^; max = 0.41 mg L^−1^). The concentrations of Ba _Total_ in the Carajás Basin, where the GCW is situated, are relatively low ($\overline{x }$ = 0.06 mg L^−1^; max = 0.24 mg L^−1^), in agreement with the observed range of concentrations of Ba _Total_ at the T site (0.05 mg L^−1^; max = 0.08 mg L^−1^). In surface water, barium can be associated with TSS by ion-exchange processes (Coffey et al., 1997), and it is originally derived from erosion and transport of soil particles, together with Fe, Al, and Mn (Sequeira et al., 2020).

#### The impacts of deforestation and urban areas on water chemistry

**Fig. 4:**
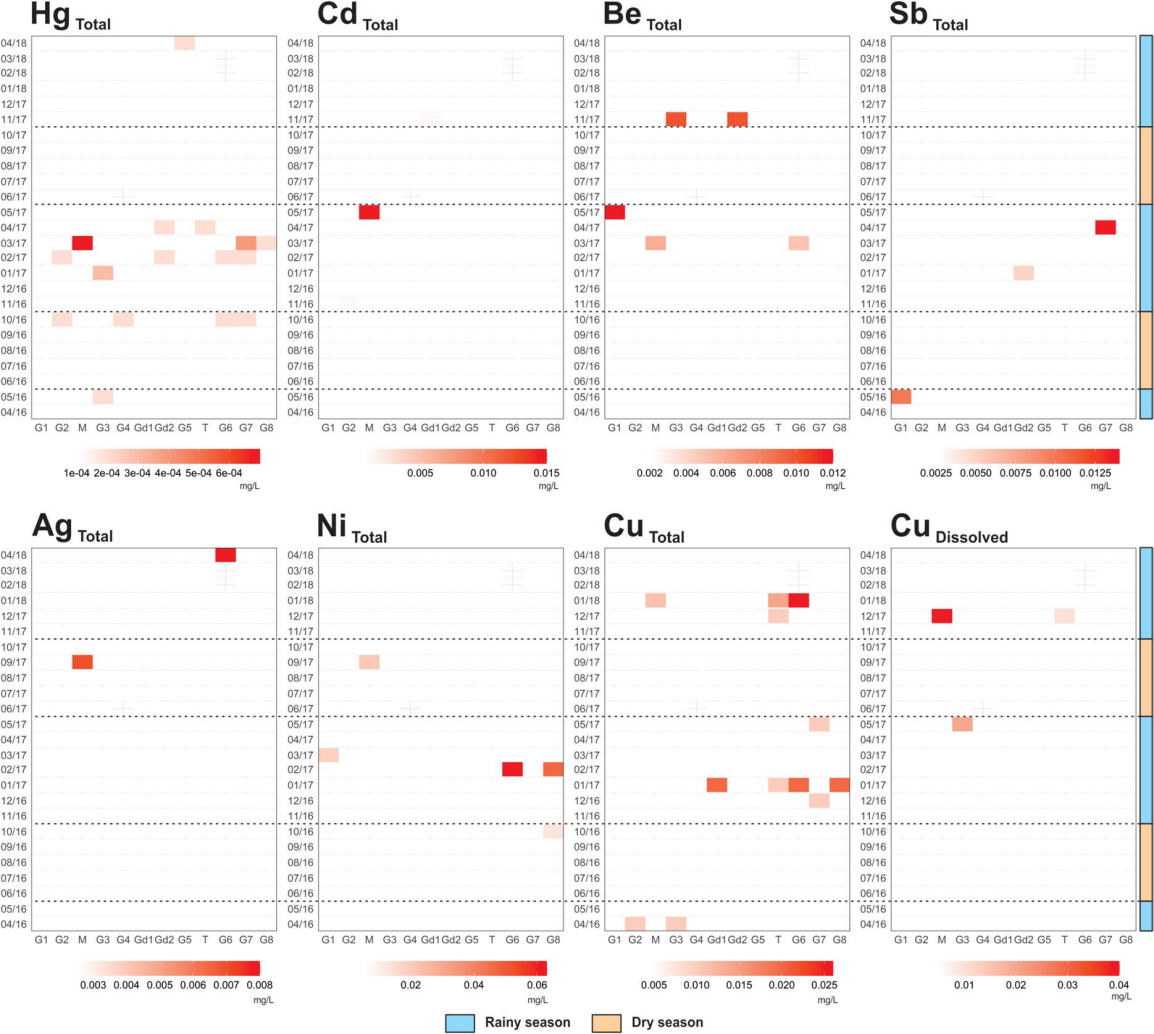
Time-series heatmaps of anomalies for selected potentially toxic elements in 12 sampling sites (Fig. 1) during 25 field campaigns conducted monthly (April 2016 to April 2018) in the Gelado Creek Watershed. “T” stands for total concentration, and “D” stands for dissolved concentration. Blank entries represent concentration below the detection limit

**Fig. 5:**
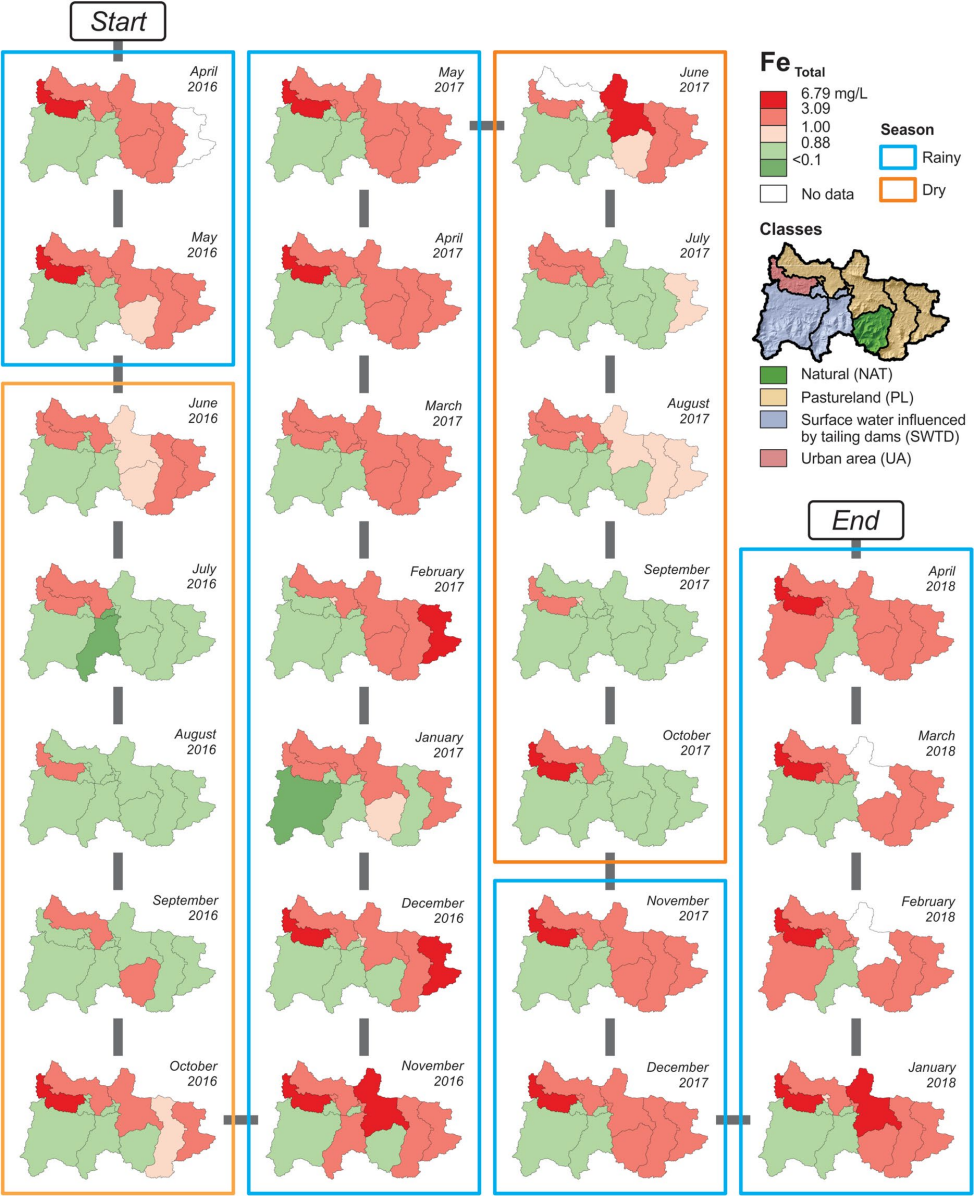
Time-series geochemical maps of Fe _Total_ in surface water of the Gelado Creek Watershed. Appendix B has information on Fe _Dissolved_, Mn _Total_, Mn _Dissolved_, Al _Dissolved_, and Ba _Total_

Time-series distribution maps of Fe _Total_ (Fig. 5) and other selected elements (Appendix B), as well as heat maps of anomalies for selected PTE (Fig. 4), indicate that the majority of the high concentrations for the above-mentioned elements were observed from the sampling sites associated with pasturelands (classified as PL, center of the LDA diagram; Fig. 3B) and urban area also associated with pastureland (classified as UA, left top corner of the LDA diagram; Fig. 3B) classes. In general, converting forested areas to pasturelands is a common characteristic of both classes. Recent studies revealed the increase of deforestation in the IGEPA and surrounding area (Théry et al., 2019), situated in the northern part of the GCW (Fig. 1B). Deforestation associated with the local intense precipitation, particularly during the rainy seasons, favors an increase in runoff and transport of solid load in streams (Salomão et al., 2023), directly influencing the concentrations of trace elements in the surface water of the Gelado Creek. Deforestation is the main factor responsible for changes (increase) in the runoff, counterbalanced by the precipitation trend in the IRW from 1973 to 2016 (Cavalcante et al., 2019). The authors show that the increase in runoff from 1973 to 2016 could be higher if a significant change (increase) in precipitation occurred. Previous studies of hydrological modeling showed that deforestation processes outside protected areas caused an increase in mean, low, and high flows in IRW rivers (Pontes et al., 2019), including the GCW. Similar outcomes have been reported globally, such as in tropical catchments in Borneo (William et al., 2025), Thailand (Pakoksung et al., 2025), and South India (Senthilkumar et al. 2018), where land-use changes led to increased runoff and altered flow regimes.

Surface water collected at the M sampling site has high concentrations of Fe _Total_ (Fig. 5), Fe _Dissolved_, Ba _Total_ (Appendix B), and TSS. Anomalous concentrations of PTE (Figs. 4, 5 and Appendix B) were also found at this site. Regarding Hg, for instance, Sahoo et al. (2023) presented evidence that the geological setting strongly controls the spatial distribution of Hg concentrations in soils and sediments of the IRW. In contrast, Hg concentrations in surface water were predominantly < 0.1 µg L^−1^. However, in comparison with this data of the entire IRW, the M site of GCW, during the campaign of March 2017 (Fig. 4), has a Hg concentration of 0.7 µg L^−1^, the highest Hg concentration in the present study. The origin of this Hg anomaly is difficult to define, but the proximity of Paulo Fonteles village (see Fig. 1B) provides a potential anthropogenic source.

#### Influences of mining tailing dam on water chemistry

**Fig. 6:**
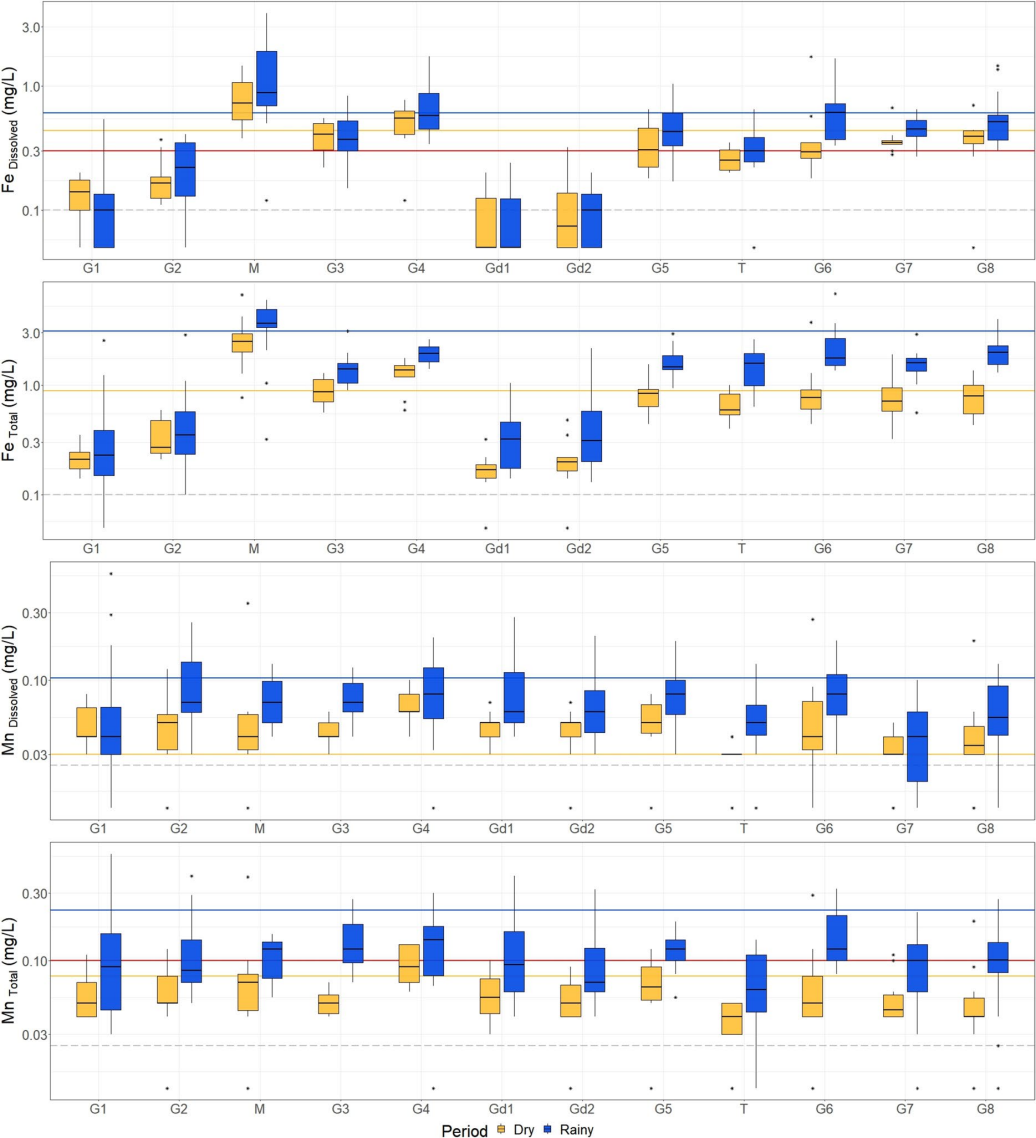
Boxplot of the Fe and Mn, both in the dissolved and total forms, in surface water samples of the 12 monitoring sites of the Gelado Creek Watershed (see Fig. 1), collected during 25 field campaigns conducted monthly (April 2016 to April 2018)

The SWMT class is represented by the G1, G2, Gd1, and Gd2 sampling sites downstream of the Gelado and Geladinho tailing dams (Fig. 1) and plotted on the right side of the LDA biplot diagram (Fig. 3B), indicating a strong influence of sulfate, Mn _Dissolved_, EC, and Turb. One hypothesis is that the tailing deposited in both dams might be a potential source of Fe to the surface water of the GCW. However, the results presented here indicate that the Fe content in these four sampling sites is, in fact, lower than the remaining sampling sites of the GCW (Fig. 6). There are two main factors contributing to this scenario: (i) the use of sediment control barriers to slow runoff velocity and filter suspended sediments from water flow and (ii) the geography (area and depth) of the Gelado and Geladinho reservoirs, which promotes the precipitation of the main minerals occurring in the tailings, including oxides and hydroxides of Fe and Mn, kaolinite [Si_2_Al_2_O_5_(OH)_4_], and quartz [SiO_2_].

In contrast, moderate to high concentrations of Mn _Dissolved_ were observed at the G1, G2, Gd1, and Gd2 sampling sites, particularly during the rainy season, from February to April 2018 (Fig. 6 and Appendix B). As presented in previous studies, the distribution of Mn _Total_ in the IRW is strongly controlled by the local lithologies (Sahoo et al., 2019). Still, the LULC also contributes to Mn distribution to a great extent (Dall’Agnol et al., 2022). Conversely, Mn _Dissolved_ shows different distribution/behavior, which suggests different controlling factors. This particular behavior can result from tailing-sediment-water interaction in the reservoirs favoring the release of Mn _Dissolved_. According to Salazar et al. (2015), the behavior of Mn at the sediment-water interface is strongly controlled by physicochemical factors (pH, redox potential, organic matter, conductivity, and organic and inorganic complexes). Results presented by Shi et al. (2021) revealed that dissolved organic carbon, DO, and pH were identified as critical factors controlling Mn speciation and Mn _Dissolved_ concentrations in water. Under these circumstances, the mobility of Mn in the Gelado reservoir (conditions also applicable to the Geladinho reservoir) can be evaluated according to the following conditions:

i) Since 1985, fine-grained Fe-rich particles have been deposited, characterizing the tailings from N4 and N5 Fe mines. The chemical composition of the Gelado tailing consists of very high Fe concentrations ($\overline{x }$ = 64.8%; range = 59.6 to 66.3%), moderate Mn ($\overline{x }$ = 0.8%; range = 0.4 to 1.3%), and low SiO_2_ ($\overline{x }$ = 1.2%; range = 0.6 to 5.8%) and Al_2_O_3_ ($\overline{x }$ = 2.0%; range = 1.2 to 4.4%) (Guimarães et al., 2020);
ii) The Gelado reservoir has a depth ranging from 2.5 to 17 m and an area of 5.6 km^2^ (Guimarães et al., 2020). It has the characteristics of a small standing-water ecosystem, a shallow lentic water body (Bolpagni et al., 2019). The tailings contain Mn compounds (mostly oxyhydroxide minerals) that should have complex interactions with water.
iii) In the Gelado reservoir, water stagnation during the dry season is common. The lentic environment and porewater conditions characteristic of the area promote, at higher depth, the decrease of DO and increase in organic matter decomposition by microbial activity (Shi et al., 2021; Tebo et al., 2004). Consequently, pH changes from near-neutral conditions to slightly acidic conditions. These allow the mobilization of Mn from the tailings and sediments to the bottom of the water column as Mn(II)_aq_, which can be eventually released through the dam bottom drain pipe.
iv) During the rainy season, the increase in precipitation and water level at the Gelado reservoir promotes the entry of oxygenated bottom currents, which favors the transport of Mn(II)aq towards the top of the water column and consequently to the dam spillway towards the Gelado Creek. This could explain the moderate to high Mn _Dissolved_ concentrations detected at G1 and G2 sampling sites. The Geladinho reservoir seems to present similar behavior (Gd1 and Gd2 sites). The influence of the precipitation in the Mn _Dissolved_ concentrations was clearly observed during the campaigns of February to April 2018 (Appendix B). These observations are consistent with other studies reporting increased Mn mobility and release under fluctuating hydrological conditions, particularly during high rainfall periods that enhance the water column mixing and sediment interaction (Davison, 1993).

In summary, moderate to low concentrations of Fe (total and dissolved) were observed in streams influenced by tailing dams, significantly lower than the previous two groups. Unusually moderate to high concentrations of dissolved Mn were observed and interpreted as an interaction of water-sediment-tailings at the bottom of the reservoirs and high precipitation events. However, this hypothesis requires further validation through robust chemical analysis and assessment of extreme precipitation, a need that has already begun to be addressed in the methodological approach presented by Silva Júnior et al. (2023).

### Determining hydrogeochemical baseline and background concentrations and their environmental application

WBM strongly supports the evaluation of hydrogeochemical background and baseline variations of trace element concentrations in surface water over time. Hydrogeochemical baseline concentrations for 23 chemical parameters (including selected metals and metalloids) of the GCW were calculated. Due to the large number of samples < DL, the calculations of geochemical baseline were not performed for Ag _Total_, As _Total_, B _Total_, Be _Total_, Cd _Total_, Co _Total_, Cr _Total_, Cu _Dissolved_, Cu _Total_, Hg _Total_, Li _Total_, Ni _Total_, Pb _Total_, Sb _Total_, Se _Total_, V _Total_, and Zn _Total_. However, for these elements, regardless of the methodological concept of determination (RA or SSA), the DL value of the analytical method of each element was assumed as its geochemical baseline (see Table 1). Extensive calculations for the threshold values of Fe _Total_, Fe _Dissolved_, Mn _Total_, Mn _Dissolved_, Al _Dissolved,_ and Ba _Total_ were performed using a variety of statistical methods (median + 2*median absolute deviation − M + 2MAD), upper tolerance limits, UTL; upper prediction limit, UPL; upper simultaneous limit, USL; and percentile-based techniques), considering both, the RA and SSA concepts (see Appendix C).

Comparatively, the different methods have advantages and disadvantages. In terms of methodological concepts, the M + 2MAD method is often considered a robust approach to identifying outliers, as it is less sensitive to outliers (Matschullat et al., 2000; Reimann & Filzmoser, 2000; Reimann et al., 2018a, 2018b). The MAD gives a strong estimate of the data deviation (specifically, for a normal distribution, the standard deviation is approximately 1.4826 times the MAD, as also presented in Reimann et al., 2018a, 2018b). The method itself is deterministic. However, the choice to use two times MAD is often justified based on probabilistic considerations to capture a similar proportion of data but in a manner more resistant to outliers. In summary, while the M + 2MAD calculation is deterministic, its rationale has roots in probabilistic considerations. The UTL and UPL methods are probabilistic, while the USL may or may not be, depending on its origin (USEPA 2022, 2023). Percentile techniques are inherently probabilistic and can be used to set thresholds or limits. The M + 2MAD, UTL, and UPL share similarities in their intent to set upper limits based on data distribution, but they employ different statistical techniques.

Percentile techniques offer a straightforward way to calculate hydrogeochemical background and baseline values. This makes their use convenient and may be the reason why some analysts and environmental agencies favor this method. Specifically, the 98th and 95th percentiles are more commonly used in environmental assessments and analyses compared to more conservative percentiles, e.g., 90th and 75th (Ander et al., 2013; Sahoo et al., 2019; Salomão et al., 2020). Furthermore, in numerous scenarios, the outcomes derived from the 98th and 95th percentile techniques align closely with results from the other complex methods discussed herein (see Appendix C). This congruence increases confidence in the values given by the 98th and 95th percentiles because they deliver results consistent with established methodologies. On the other hand, the 90th and 75th percentiles, often considered a reference for determining background values, particularly by the National Environment Council of Brazil (CONAMA - Conselho Nacional do Meio Ambiente 2009), are not suitable for this purpose, mainly due to the resulting conservative values obtained (Salomão et al., 2020), which are counter-productive and costly for risk analysis (Reimann et al., 2005, 2011). Moreover, percentile techniques operate on predefined thresholds to establish what is considered “anomalous.” This practice raises scientific criticisms (Reimann, et al., 2018a, 2018b; Salomão et al., 2020) because it imposes a fixed proportion of samples to be categorized as outliers (Sauvaget et al., 2020) without taking into consideration the inherent characteristics and distribution of the dataset.

**Fig. 7:**
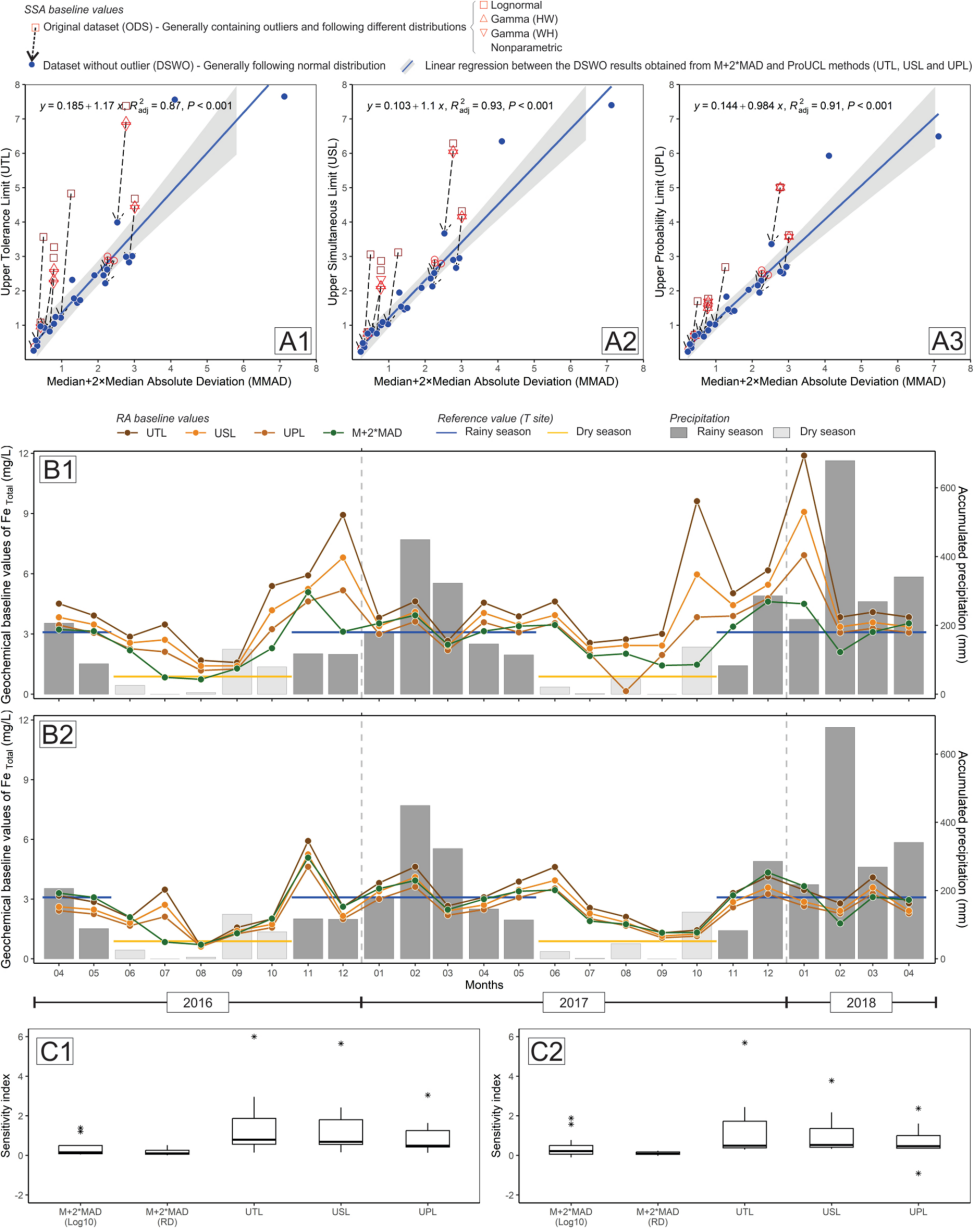
Comparison of geochemical baseline results for Fe _Total_, considering site-specific (SSA) and regional (RA) assessments. SSA: comparison of results from M + 2MAD with UTL (A1), USL (A2), and UPL (A3), considering the subset of data (original dataset – ODS or dataset without outliers – DSWO) and data distribution (normal, lognormal, gamma, or nonparametric). The vector indicates the change in the final result after removing the outlier, possibly associated with the shift in data distribution. RA: geochemical baseline value of each field campaign (considering all 12 sampling sites) from April 2016 to April 2018, considering the ODS (B1) and the DSWO (B2), in comparison with the accumulated precipitation (mm) record of the area. Hydrogeochemical background values determined for the T sampling site are horizontal lines for the rainy (blue) and dry (yellow) seasons. Boxplot of the sensitivity index for each statistical method, considering the normalized difference of the results obtained from ODS and DSWO for the SSA (C1) and RA (C2)

ProUCL offers an extensive list of possible options for consideration (see Appendix C), which includes UTL, USL, and UPL, and the choice of method can vary significantly based on the data distribution, whether it is normal, lognormal, gamma, or nonparametric (see Appendix C). Numerous options can often lead to choose overload, complicating the tasks of environmental analysts. Particularly in the absence of statistical experts, making informed and assertive decisions challenging. In contrast, the M + 2MAD method is simple and efficient in calculating background and baseline values. Its straightforward characteristic has led to its widespread adoption (Reimann et al., 2018a, 2018b; Reimann & Caritat, 2017; Sahoo et al., 2019, 2020; Salomão et al., 2020, 2021), not only attesting its reliability but also facilitating agreement across studies. When comparing the results obtained from these different statistical methods, the better performance of M + 2MAD in both approaches, SSA (Fig. 7A1, A2, and A3) and RA (Fig. 7B1 and B2), is apparent, in comparison to the other methods. In addition, the M + 2MAD (Log_10_ and RD) gave a lower sensitivity index comparatively to UTL, USL, and UPL (Fig. 7C1 and C2), indicating better performance when considering the presence of outliers in the data set before calculating background values. Similar results have been reported elsewhere in comparison to other methods (Reimann et al., 2005; Sauvaget et al., 2020). Among the different statistical methods used to derive hydrogeochemical background and baseline values, the M + 2MAD provided the most consistent results, making it a preferred choice for many researchers.

The estimation of the geochemical baseline based on the RA approach was important for the identification of contamination sources. It systematically identified the M site’s catchment area (Fig. 1) as a primary contamination source in the GCW. Most importantly, this approach provided a strong graphical output to understand the influence of the precipitation in the geochemical baseline concentrations in a small-scale watershed (Fig. 7B). Consequently, the need to consider the seasonality for baseline/background calculations is clearly demonstrated. In addition, the RA results can be used as baseline values of the GCW at the time of the field campaign, which can be used as reference values for further monitoring but not as a hydrogeochemical background threshold limit. The main reason for this is the fact that most of the sampling sites are actually influenced by anthropogenic activities. For instance, G1, G2, Gd1, and Gd2 revealed low concentrations of Fe and some PTE. In contrast, the stream water collected at the M sampling site is strongly contaminated and exerted a direct influence on the chemistry of the water collected in the sampling sites located downstream (G3 to G8). These sites, including the M site, are also strongly influenced by deforestation. Similar results were presented and discussed for the IRW (Dall’Agnol et al., 2022; Sahoo et al., 2019). Regarding naturally occurring hydrogeochemical background values, the T sampling site is the only monitoring point that approaches pristine conditions in the area which can truly identify natural conditions. This highlights the importance of deriving background values based on the SSA approach (Appendix C) in pristine areas. Figure 6 demonstrates the importance of considering the results calculated for the T site as a background value. The Fe concentrations at the M sampling site are clearly above the limits adopted as a reference, which indicates, once again, the fact that these are anomalous concentrations related to a local contamination source, in agreement with the previous discussion herein. On the other hand, the behavior of Fe _Total_ and Fe _Dissolved_ in G1, G2, Gd1, and Gd2 (all sampling points related to the dams) is entirely distinct from that observed at the M site. This demonstrates that the water running out of the reservoirs should not be considered responsible for Fe contamination in the GCW; however, particular attention should be given to Mn during the rainy seasons, which has shown moderate to high dissolved concentrations downstream of the dams (Appendix B.2 and B.3).

Table 2 summarizes the hydrogeochemical background threshold values of the T sampling site and compares them with values reported in other studies, as well as with regulatory limits established by the Brazilian environmental regulation agency (CONAMA, 2005) and the World Health Organization (WHO, 2008). A previous study conducted in the Carajás region (Teixeira et al., 2020), precisely at preserved areas of the N3 plateau and at the southwest extension of the N4 Fe mine (outside the GCW; Fig. 1B), revealed relatively low concentrations of Fe (total and dissolved), but high concentrations of Mn (total and dissolved) in surface water, in comparison to the T sampling site of GCW (Table 2). This variance may be linked to the occurrence of Paleoproterozoic clastic and chemical sedimentary rocks hosting manganese deposits (Araújo & Nogueira, 2019; Nogueira et al., 1995) or even disseminated occurrences of Mn related to the Neoarchean metavolcano-sedimentary rocks (Klein, 2005) in the area studied by Teixeira et al. (2020). Natural hydrogeochemical background variations concerning the geological setting and LULC are regularly reported (Table 2): (i) watershed influenced by granites and metasedimentary rocks of the Mondego River in Portugal (Sequeira et al., 2020); (ii) the Loire River Basin in France (Gassama et al., 2021); (iii) surface water of Europe (De Vos et al., 2006).

**Table 2:** Proposed hydrogeochemical background concentrations for Fe _Total_, Fe _Dissolved_, Mn _Total_, Mn _Dissolved_, Al _Dissolved,_ and Ba _Total_ in surface water of the Gelado Creek Watershed (GCW). See Appendix C for detailed information

Study area or regulation guidelines	Source	Sampling strategy	Filtration	Geochemical background method	Season	Fe _T_	Fe_D_	Mn_T_	Mn_D_	Al_D_	Ba_T_
Gelado Creek Watershed – natural area (T sampling site)	Present study	SSA	0.45 µm	UTL	Rainy	2.99	0.73	0.19	0.14	0.25	0.10
					Dry	1.24	0.43	0.08	0.04	0.18	0.06
				MMAD	Rainy	3.09	0.60	0.23	0.23	< 0.05	0.10
					Dry	0.88	0.44	0.08	0.08	0.21	0.04
Vicinity of N3 plateau – Fe deposit	Teixeira et al. (2020)	SSA	0.45 µm	USL	-	0.92	0.32	0.79	0.79	0.127	-
				MMAD	-	0.22	-	-	-	-	-
Vicinity of N4WSul plateau – Fe mine	Teixeira et al. (2020)	SSA	0.45 µm	USL	-	1.3	0.45	1.02	0.84	0.23	-
				MMAD	-	1.14	0.62	0.13	0.05	0.77	-
Itacaiúnas River Watershed	Sahoo et al. (2019)	RA	0.45 µm	MMAD	Rainy	7.76	-	0.91	-	-	0.30
					Dry	7.59	-	1.55	-	-	0.37
Mondego River – metasedimentary rocks (RC1)	Sequeira et al. (2020)	SSA		MMAD	-	-	0.13	-	0.0125	0.03	-
Mondego River – granites (CAV site)	Sequeira et al. (2020)	SSA		MMAD	-	-	0.20	-	0.041	0.03	-
FOREGS – Geochemical Atlas of Europe	De Vos et al. (2006)	RA	0.45 µm	Median	-		0.067		0.0159	0.0177	-
Amazon River	Compiled from (Gailladert et al. (2003)	SSA	0.2 µm	Mean value	-	-	0.043	-	0.00331	0.0062	-
Negro River	Compiled from Gailladert et al. ( 2003)	SSA	0.2 µm	Mean value	-	-	0.117	-	0.00735	0.097	-
Solimões River	Compiled from Gailladert et al. (2003)	SSA	0.2 µm	Mean value	-	-	0.351	-	0.01456	0.1714	-
Global Water	Compiled from Gailladert et al. (2003)	SSA	0.2 µm	Mean value	-	-	0.066	-	0.034	0.032	-
Global Water	Ivanov (1996)	-			-	-	0.04	-	0.010	0.160	-
CONAMA N°357/2005- Fresh water - Class 1 and 2	CONAMA (2005)	-	0.45 µm	-	-	-	0.3	0.1	-	0.1	0.7
CONAMA N°357/2005- Fresh water - Class 3	CONAMA (2005)	-	0.45 µm	-	-	-	5.0	0.5	-	0.2	1.0
Guidelines for drinking-water quality	WHO (2008)					-	-	0.3	0.4	-	0.2

‘*’ CONAMA (2005) fresh water

The above-mentioned studies have shown that some elements have background concentrations higher than regulatory limits for reasons unrelated to human activity but directly related to the geological setting, as observed in the present study (Fig. 6). It is unreasonable to attribute reference values lower than background values in diverse geological places and ecosystems, and the unique characteristics of each region must be considered for proper classification. These findings can assist in defining an accurate picture of a watershed and the spatial distribution of anomalies, which are of great relevance to assessing future anthropogenic impacts.

It is important to acknowledge that the definition of the “dissolved” fraction, based on 0.45 µm filtration, presents well-documented limitations. While widely adopted in environmental monitoring and regulatory frameworks (e.g., APHA 2017; CONAMA, 2005), this threshold does not accurately separate truly dissolved species (< 2 nm) from colloidal particles (2 nm to 0.45 µm), which can play a significant role in metal transport and geochemical behavior. The inclusion of colloids may lead to overestimation of the dissolved fraction for particle-reactive elements such as Fe, Mn, and Al, while simultaneously masking dissolved concentrations due to sorption processes (Stumm and Morgan, 1996; Filella et al.,^2006^). This is particularly relevant when comparing datasets from different studies that employ varying filtration methods, as reflected in the variability observed in Table 2. Although this study followed the standard 0.45 µm protocol, we recognize this methodological constraint and recommend that future investigations incorporate ultrafiltration or advanced speciation techniques to better differentiate dissolved, colloidal, and particulate phases, thereby improving the accuracy of hydrogeochemical background assessments.

## Conclusion and future perspectives

The integrated assessment applied herein aids in evaluating the multifactorial influences on hydrogeochemical baseline concentrations and identifying contamination sources in a watershed located in the eastern Amazon rainforest. Based on the results and discussions presented in this study, the following conclusions can be drawn about the GCW:

- Time-series maps and multivariate hydrogeochemical patterns revealed four main groups of catchments, mainly influenced by the LULC, one influenced by preserved area covered by forest, where human impact can be considered insignificant, and three other groups influenced by pasturelands, urban areas, and mining tailing dams.
- The highest concentrations of Fe and selected PTE were observed at the M sampling site with integrated influence from an urban area and pasturelands in the GCW. High concentrations were also observed in the catchment areas with a predominance of pastureland due to the increase of runoff effect as a consequence of bare soil exposure. Future studies based on high-resolution remote sensing are needed to accurately map out small-scale rural areas and villages, as they can be potential sources of alteration of surface water in the study area.
- Moderate to low concentrations of Fe (total and dissolved) were observed in streams influenced by tailing dam, significantly lower in comparison with the previous two groups. Unusually moderate to high concentrations of dissolved Mn were observed and interpreted as an interaction of water-sediment-tailings at the bottom of the dam reservoirs and high precipitation events, but that needs a more robust assessment of chemical analysis and precipitation extremes to validate this hypothesis.
- Two approaches were implemented for investigation of hydrogeochemical background and baseline values: (i) RA was important for identifying contamination sources and understanding the influence of precipitation on hydrogeochemical baseline concentrations. It clearly demonstrated the need to consider seasonality before calculating background values. In addition, it was concluded that the RA results could be used as baseline values of the GCW at the time of the field campaign, important for detailed environmental monitoring, but not as a hydrogeochemical background threshold limit; (ii) SSA allows the establishment of hydrogeochemical background values, based on metal concentrations observed at the T sampling site, interpreted as representative of pristine conditions in the study area. Among the different statistical methods used to derive hydrogeochemical background values, the M + 2MAD gave the most consistent results. In developing geochemical studies in a watershed, exploring the geospatial characteristics of the watershed under investigation is highly recommended prior to solely calculating reference values.
- RA provides results into a wider perspective by investigating larger areas and periods, thus complementing SSA and giving a macroscopic view of geochemical variations. SSA complements RA by delivering detailed and localized data that may be crucial for highlighting specific sources of contamination and understanding local geochemical processes. These values derived from both RA and SSA can be applied to environmental management in the provision of benchmarks against which the impact of anthropogenic activities should be monitored and assessed. These values are essential in setting regulatory standards that guide remediation efforts and policy decisions in protecting and managing water resources.

Overall, this integrated study effectively identified contamination sources in a watershed of the southeastern Amazon. It represents a contribution to understanding the Carajás region’s environmental geochemistry. Future studies will be conducted in the GCW region by the ITV scientific team, aiming to monitor and evaluate the geochemical influence of the Gelado tailing dam on the surface water of Gelado Creek. To evaluate the safety and risk management related to chemicals in urban areas, rural communities, and protected areas, future studies should incorporate (i) physical and chemical assessments of water quality and sediments; (ii) groundwater investigations, considering that groundwater-surface water interactions play a critical role in hydrogeochemical processes and strongly influence surface water composition; (iii) microbiological analyses, including DNA-based approaches, to better understand microbial-driven geochemical transformations; and (iv) the evaluation of atmospheric deposition, a major but often overlooked pathway for metal and contaminant input into aquatic systems. Remote and in situ environmental monitoring should be encouraged within the Amazon region to provide a robust risk assessment, addressing real impacts on the ecosystem.

## Electronic supplementary material

Below is the link to the electronic supplementary material.

Supplementary Material 1

Supplementary Material 1

## Data Availability

Data will be made available upon request.
