# Supplementary material for: Effect of land use, geology, and seasonality on hydrogeochemical baseline variations in a watershed impacted by human activities in the eastern Amazon
Source: Environ Monit Assess. 2025 Sep 22;197(10):1138. doi: 10.1007/s10661-025-14565-7 (PMC12454543; doi:10.1007/s10661-025-14565-7)
Supplement: Supplementary file 1 — Supplementary Material 1 [file 10661_2025_14565_MOESM1_ESM.docx]

**Appendices**

**Manuscript Title:** Effect of land use, geology, and climate seasonality on hydrogeochemical baseline variations in a watershed impacted by human activities in the eastern Amazon

**Authors:** Gabriel Negreiros Salomão*, Roberto Dall’Agnol, Gabriel Soares de Almeida, Rafael Tarantino Amarante, Glariston Miranda Mello, Renato Oliveira da Silva Júnior, José Tasso Felix Guimarães, Marcio Sousa da Silva, Danieli Mara Ferreira, Paulo Rógenes Monteiro Pontes, Prafulla Kumar Sahoo, Eduardo Duarte Marques and Emmanoel Vieira da Silva Filho

***Corresponding author email:** Gabriel.salomao@itv.org

**Summary**

[Appendix A – Geochemical and geospatial data processing and exploratory analysis 2](#_Toc152228040)

[Appendix B – Time-series geochemical maps of Fe _Dissolved_, Mn _Total_, Mn _Dissolved_, Al _Dissolved_, and Ba _Total_ in surface water of the Gelado Creek Watershed 7](#_Toc152228041)

# Appendix A – Geochemical and geospatial data processing and exploratory analysis

**Data treatment and statistical analysis**: The quality of the data was examined in the laboratory by using control samples (laboratory-fortified blank) with measurable recovery between 80 and 120%. Only parameters with acceptable quality and sampling sites with reasonable time series record were considered for this study (Table 1). Geochemical censored data (<LLD) was replaced by LLD/2. Descriptive statistics, time series analysis, regression analysis and matrix correlations were performed to better understand the data set in the concept of exploratory data analysis and, consequently, to select relevant chemical parameters for further investigation. Boxplots, histograms, Q-Q plots, normal probability plots were used to better represent and compare results. Data analysis was performed using the R programming language in RStudio (RStudio Team, 2020), which is associated with a combination of R packages (Lorenz and Diekoff, 2017; Wickham, 2016).

**Geospatial characterization**: The catchment area, kwon as the unit of area which discharges runoff water at particular point called as outlet, of each 12 sampling sites (Figure 1) was generated by using a 30-meter spatial resolution digital elevation model, acquired by the “DAICHI” Advanced Land Observing Satellite (ALOS) launched by the Japan Aerospace Exploration Agency (JAXA). The sampling site catchment area was plotted over the LULC (Figure 1B) and geological layers (Figure 1C) for the calculation of spatial area (km^2^) contribution of each class at the sampling site catchment, by using zonal statistics tool in a geoprocessing environment, using ArcGIS PRO software (Esri, 2023). These results were then transformed to percentage of contribution (Table A.1) and submitted to a principal component analysis (PCA), computed using ‘stats’ package (R Core Team, 2022) in RStudio (RStudio Team, 2020). PCA loadings and scores were extracted to construct the scatter plot used for the identification and grouping of similar catchment areas in terms of geospatial characteristics (LULC and geology).

Table A.1 - Percentage share of land use and land cover (LULC) and geology classes of each sampling point catchment area.

| **Surface water monitoring sites** | | **G1** | **G2** | **M** | **G3** | **G4** | **Gd1** | **Gd2** | **G5** | **T** | **G6** | **G7** | **G8** |
| --- | --- | --- | --- | --- | --- | --- | --- | --- | --- | --- | --- | --- | --- |
| **Catchment Area (km^2^)** | | 132.5 | 138.9 | 32.3 | 172.9 | 230.9 | 58.8 | 62.7 | 294.9 | 52.2 | 419.0 | 490.5 | 543.6 |
| **LULC (%)** | **Forest** | 73.2 | 72.1 | 50.5 | 67.8 | 58.5 | 76.0 | 75.8 | 62.2 | 99.3 | 65.7 | 68.1 | 67.3 |
|  | **Pastureland** | 3.7 | 5.8 | 48.7 | 14.3 | 28.0 | 4.4 | 5.8 | 23.4 | 0.7 | 24.0 | 23.1 | 24.7 |
|  | **Mining** | 16.1 | 15.4 |  | 12.4 | 9.3 | 18.4 | 17.3 | 10.9 |  | 7.7 | 6.6 | 5.9 |
|  | **Urban Area** |  |  | 0.7 | 0.1 | 0.1 |  |  | 0.1 |  | 0.2 | 0.1 | 0.1 |
|  | **Water** | 4.3 | 4.2 |  | 3.3 | 2.6 | 1.0 | 1.0 | 2.2 |  | 1.6 | 1.4 | 1.3 |
|  | **Montana Savanna** | 2.7 | 2.6 |  | 2.1 | 1.6 | 0.1 | 0.1 | 1.2 |  | 0.9 | 0.7 | 0.7 |

(Continued)

Table A.1 - Percentage share of land use and land cover (LULC) and geology classes of each sampling point catchment area.

| **Surface water monitoring sites** | | **G1** | **G2** | **M** | **G3** | **G4** | **Gd1** | **Gd2** | **G5** | **T** | **G6** | **G7** | **G8** |
| --- | --- | --- | --- | --- | --- | --- | --- | --- | --- | --- | --- | --- | --- |
| **Catchment Area (km^2^)** | | 132.5 | 138.9 | 32.3 | 172.9 | 230.9 | 58.8 | 62.7 | 294.9 | 52.2 | 419.0 | 490.5 | 543.6 |
| **Geology (%)** | **Paleogene lateritic cover**  **(PLC)** | 3.4 | 3.2 |  | 2.6 | 1.9 | 25.2 | 23.7 | 6.5 | 11.1 | 6.1 | 5.7 | 5.2 |
|  | **Paleoproterozoic anorogenic granite (PAG)** |  |  |  |  |  |  |  |  |  |  |  | 0.5 |
|  | **Paleoproterozoic sedimentary rocks (PSR)** | 5.6 | 5.3 |  | 4.3 | 3.2 |  |  | 2.5 |  | 1.8 | 1.5 | 1.4 |
|  | **Neoarchean A-type like granite (NAG)** | 25.8 | 28.3 | 99.4 | 42.3 | 55.4 | 16.1 | 19.5 | 48.1 |  | 43.1 | 38.8 | 38.5 |
|  | **Neoarchean mafic to felsic metavolcano-sedimentary rocks (NMVS)** | 65.3 | 63.1 | 0.6 | 50.8 | 39.5 | 58.7 | 56.8 | 42.9 | 88.9 | 49.1 | 53.9 | 54.5 |

(Concluded)

**Multivariate hydrogeochemical patters**: This study applied a series of multivariate statistics techniques to evaluate multitemporal and spatial patterns of hydrogeochemical parameters in surface water of the GCW. The preprocessing dataset (centering and scaling) was submitted to a classic linear discriminant analysis (LDA) in order to determine the discriminant function, which allows the optimal separation of group of samples based on hydrogeochemical data of the classes derived from the PCA classification (description given above). LDA was computed using ‘MASS’ package (Venables and Ripley, 2002) in Rstudio. Linear discriminant functions were constructed using the unstandardized discriminant coefficient ($b_{i}$; Table A.2) and the measurement of the water quality parameter ($C_{i}$), following the eq. (1).

$$LD=b_{1}C_{1}+b_{2}C_{2}+\ldots+b_{i}C_{i} (1)$$

Table A.2 – Water quality parameters computed in linear discriminant analysis, considering the preprocessing transformation of the geochemical data (centering and scaling). The calculated discriminant coefficients for the first two functions (LD1 and LD2) are presented.

| **Parameters** | **Preprocessing transformation** | | **Coefficients of linear discriminants** | |
| --- | --- | --- | --- | --- |
|  | $\bar{\boldsymbol{x}}$ | **s** | **LD1** | **LD2** |
| Sulfate (SO_4_^2-^) | 2.74 | 1.60 | 1.212** | 0.783** |
| Mn _Dissolved_ | 0.067 | 0.060 | 0.618** | -0.559** |
| Electrical Conductivity (EC) | 63 | 42 | 0.241* | 0.081 |
| Turbidity (Turb) | 15.35 | 13.54 | 0.216* | -0.158 |
| Cu _Total_ | 0.005 | 0.002 | 0.207* | -0.330* |
| Nitrate (NO_3_^-^) | 0.55 | 1.18 | 0.137 | 0.047 |
| Cr _Total_ | 0.006 | 0.007 | 0.084 | -0.206* |
| Al _Dissolved_ | 0.08 | 0.08 | -0.137 | -0.033 |
| Mn _Total_ | 0.100 | 0.080 | -0.255* | 0.170 |
| Total suspended solids (TSS) | 14.8 | 16.6 | -0.297* | 0.012 |
| Hg _Total_ | 0.00011 | 0.00005 | -0.323* | 0.126 |
| Fe _Total_ | 1.24 | 1.15 | -0.347* | 0.998** |
| Ba _Total_ | 0.04 | 0.02 | -0.357* | -0.276* |
| Chloride (Cl^-^) | 1.98 | 0.72 | -0.451** | -0.858** |
| Fe _Dissolved_ | 0.40 | 0.34 | -0.457** | 0.743** |

Note: $\bar{\boldsymbol{x}}$ = mean; s = standard deviation; ‘**’ Parameter with very high relevance; ‘*’ Parameter with moderate relevance;

**Determination of geochemical baseline concentration values:** Geochemical threshold values were determined by integrating a variety of robust statistical methods, which are widely applied in the literature (Ander et al., 2013; Reimann et al., 2018, 2005; Reimann and Caritat, 2017, 2005; Sahoo et al., 2020, 2019; Salomão et al., 2020; Teixeira et al., 2020): median + 2*median absolute deviation (M+2MAD), ProUCL statistically derived baseline values (upper tolerance limits - UTL; upper prediction limit - UPL; upper simultaneous limit - USL), and percentile-based techniques (98^th^, 95^th^, 90^th^ and 75^th^). The description and math equations for each method can be found elsewhere (Ander et al., 2013; Matschullat et al., 2000; Reimann and Caritat, 2005). The above-mentioned methods were calculated in RStudio (RStudio Team, 2020) and in ProUCL software (United States Environmental Protection Agency - USEPA, 2022). Two different approaches were implemented in this study: i) Site-specific assessment (SSA), which consists in calculating the background or baseline values of an element or a chemical compound for a given sampling site, considering multiple continuous samplings in a time series record; ii) Regional assessment (RA), which considers the data collected of multiple sampling sites in the region of interest, from a specific sampling campaign. The seasonality (rainy and dry periods) of the study area was considered for the data processing. Two datasets were considered for calculation, the original dataset (ODS) and the dataset without outliers (DSWO). The sensitivity index, the normalized difference of the baseline values obtained from ODS and DSWO, was calculated for each statistical method used herein to evaluate the response of the presence of outliers on the geochemical baseline values. For each data population, the distribution (normal – N; lognormal – LN; gamma – G; non-parametric – NP; or degenerative probability distribution - DPD) was tested by using goodness-of-fit test (GoF). When applicable, cases where the data population follows more than one distribution were also considered. The Dixon’s outlier test and the GoF test were performed in the ProUCL software (United States Environmental Protection Agency - USEPA, 2022).

**References**

Ander, E.L., Johnson, C.C., Cave, M.R., Palumbo-Roe, B., Nathanail, C.P., Lark, R.M., 2013. Methodology for the determination of normal background concentrations of contaminants in English soil. Science of the Total Environment 454–455, 604–618. https://doi.org/10.1016/j.scitotenv.2013.03.005

Esri, 2023. ArcGIS for Desktop.

Lorenz, D.L., Diekoff, A.L., 2017. smwrGraphs—An R package for graphing hydrologic data, version 1.1.2.

Matschullat, J., Ottenstein, R., Reimann, C., 2000. Geochemical background - can we calculate it? Environmental Geology 39, 990–1000. https://doi.org/10.1007/s002549900084

R Core Team, 2022. R: A language and environment for statistical computing. R foundation for statistical computing [WWW Document]. URL https://www.r-project.org/

Reimann, C., Caritat, P., 2017. Establishing geochemical background variation and threshold values for 59 elements in Australian surface soil. Science of the Total Environment 578, 633–648. https://doi.org/10.1016/j.scitotenv.2016.11.010

Reimann, C., Caritat, P., 2005. Distinguishing between natural and anthropogenic sources for elements in the environment: Regional geochemical surveys versus enrichment factors. Science of the Total Environment 337, 91–107. https://doi.org/10.1016/j.scitotenv.2004.06.011

Reimann, C., Fabian, K., Birke, M., Filzmoser, P., Demetriades, A., Négrel, P., Oorts, K., Matschullat, J., de Caritat, P., Albanese, S., Anderson, M., Baritz, R., Batista, M.J., Bel-Ian, A., Cicchella, D., De Vivo, B., De Vos, W., Dinelli, E., Ďuriš, M., Dusza-Dobek, A., Eggen, O.A., Eklund, M., Ernsten, V., Flight, D.M.A., Forrester, S., Fügedi, U., Gilucis, A., Gosar, M., Gregorauskiene, V., De Groot, W., Gulan, A., Halamić, J., Haslinger, E., Hayoz, P., Hoogewerff, J., Hrvatovic, H., Husnjak, S., Jähne-Klingberg, F., Janik, L., Jordan, G., Kaminari, M., Kirby, J., Klos, V., Kwećko, P., Kuti, L., Ladenberger, A., Lima, A., Locutura, J., Lucivjansky, P., Mann, A., Mackovych, D., McLaughlin, M., Malyuk, B.I., Maquil, R., Meuli, R.G., Mol, G., O’Connor, P., Ottesen, R.T., Pasnieczna, A., Petersell, V., Pfleiderer, S., Poňavič, M., Prazeres, C., Radusinović, S., Rauch, U., Salpeteur, I., Scanlon, R., Schedl, A., Scheib, A., Schoeters, I., Šefčik, P., Sellersjö, E., Slaninka, I., Soriano-Disla, J.M., Šorša, A., Svrkota, R., Stafilov, T., Tarvainen, T., Tendavilov, V., Valera, P., Verougstraete, V., Vidojević, D., Zissimos, A., Zomeni, Z., Sadeghi, M., 2018. GEMAS: Establishing geochemical background and threshold for 53 chemical elements in European agricultural soil. Applied Geochemistry 88, 302–318. https://doi.org/10.1016/j.apgeochem.2017.01.021

Reimann, C., Filzmoser, P., Garrett, R.G., 2005. Background and threshold: Critical comparison of methods of determination. Science of the Total Environment 346, 1–16. https://doi.org/10.1016/j.scitotenv.2004.11.023

RStudio Team, 2020. RStudio: Integrated Development for R.

Sahoo, P.K., Dall’Agnol, R., Salomão, G.N., Junior, J. da S.F., Silva, M.S., Souza Filho, P.W.M., Costa, M.L., Angélica, R.S., Medeiros Filho, C.A., Costa, M.F., Guilherme, L.R.G., Siqueira, J.O., 2020. Regional-scale mapping for determining geochemical background values in soils of the Itacaiúnas River Basin, Brazil: The use of compositional data analysis (CoDA). Geoderma 376, 114504. https://doi.org/10.1016/j.geoderma.2020.114504

Sahoo, P.K., Dall’Agnol, R., Salomão, G.N., Silva Ferreira Junior, J., Silva, M.S., Souza Filho, P.W.M., Powell, M.A., Angélica, R.S., Pontes, P.R., Costa, M.F., Siqueira, J.O., 2019. High resolution hydrogeochemical survey and estimation of baseline concentrations of trace elements in surface water of the Itacaiúnas River Basin, southeastern Amazonia: Implication for environmental studies. J Geochem Explor 205, 106321. https://doi.org/10.1016/j.gexplo.2019.06.003

Salomão, G.N., Dall’Agnol, R., Sahoo, P.K., Angélica, R.S., Medeiros Filho, C.A., Ferreira Júnior, J. da S., Sousa da Silva, M., Souza Filho, P.W.M. e, Nascimento Junior, W. da R., Costa, M.F., Guilherme, L.R.G., Siqueira, J.O. de, 2020. Geochemical mapping in stream sediments of the Carajás Mineral Province: Background values for the Itacaiúnas River watershed, Brazil. Applied Geochemistry 118, 104608. https://doi.org/10.1016/j.apgeochem.2020.104608

Teixeira, S.S., Dall’Agnol, R., Sahoo, P.K., Salomão, G.N., Guimarães, J.T.F., Costa, M., 2020. Water chemistry and estimation of background levels of elements in surface water bodies from a protected area in the vicinity of Fe deposits, Southeastern Amazon. Environ Forensics 21, 176–194. https://doi.org/10.1080/15275922.2020.1728436

United States Environmental Protection Agency - USEPA, 2022. ProUCL: Statistical Software for Environmental Applications for Data Sets with and without Nondetect Observations [WWW Document]. URL https://www.epa.gov/land-research/proucl-software (accessed 7.12.23).

Venables, W.N., Ripley, B.D., 2002. Modern Applied Statistics with S, 4th ed. Springer, New York.

Wickham, H., 2016. ggplot2: Elegant Graphics for Data Analysis [WWW Document]. Springer-Verlag New York. URL https://ggplot2.tidyverse.org (accessed 11.29.22).

# Appendix B – Time-series geochemical maps of Fe _Dissolved_, Mn _Total_, Mn _Dissolved_, Al _Dissolved_, and Ba _Total_ in surface water of the Gelado Creek Watershed


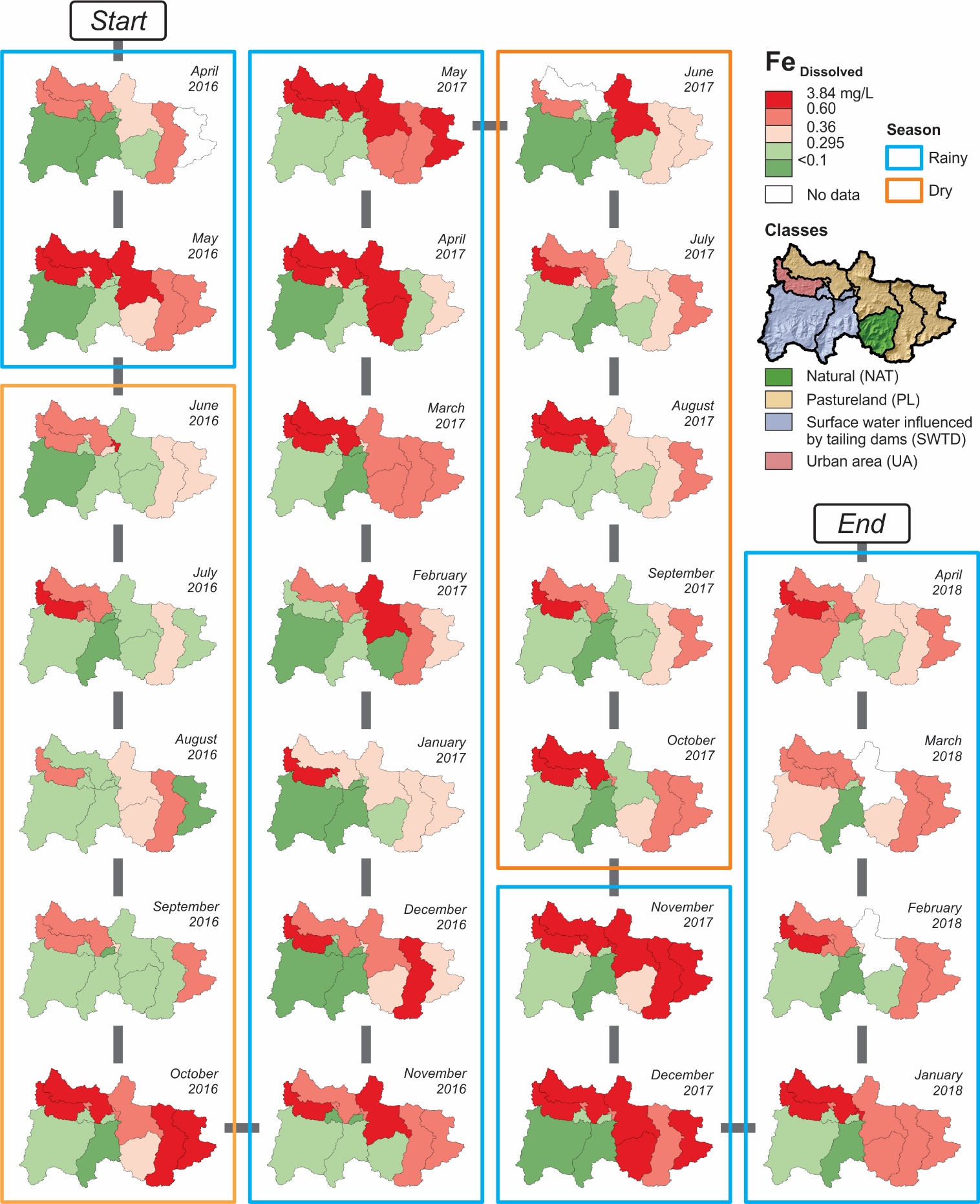


Appendix B.1. Time-series geochemical maps of Fe _Dissolved_ in surface water of the Gelado Creek Watershed.


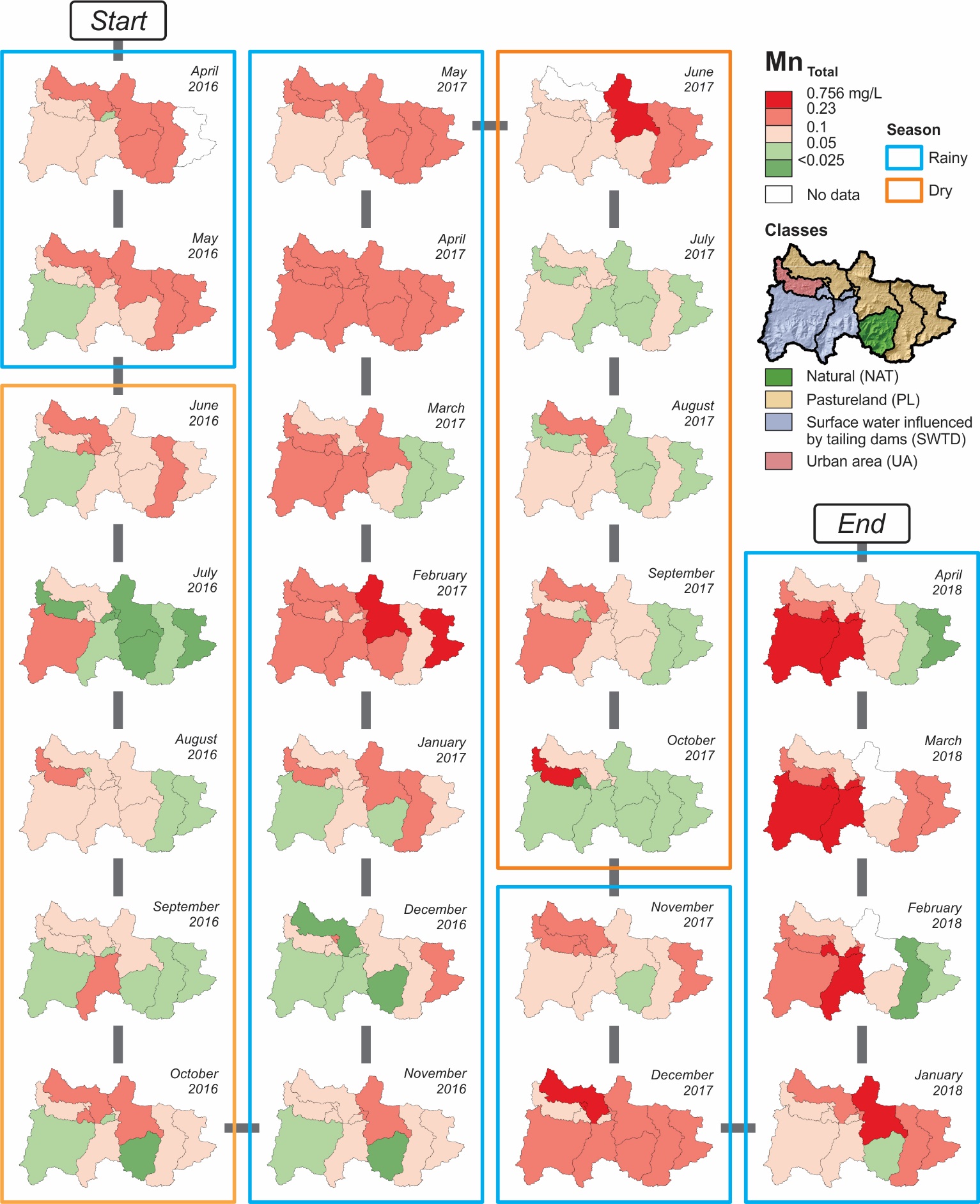


Appendix B.2. Time-series geochemical maps of Mn _Total_ in surface water of the Gelado Creek Watershed.


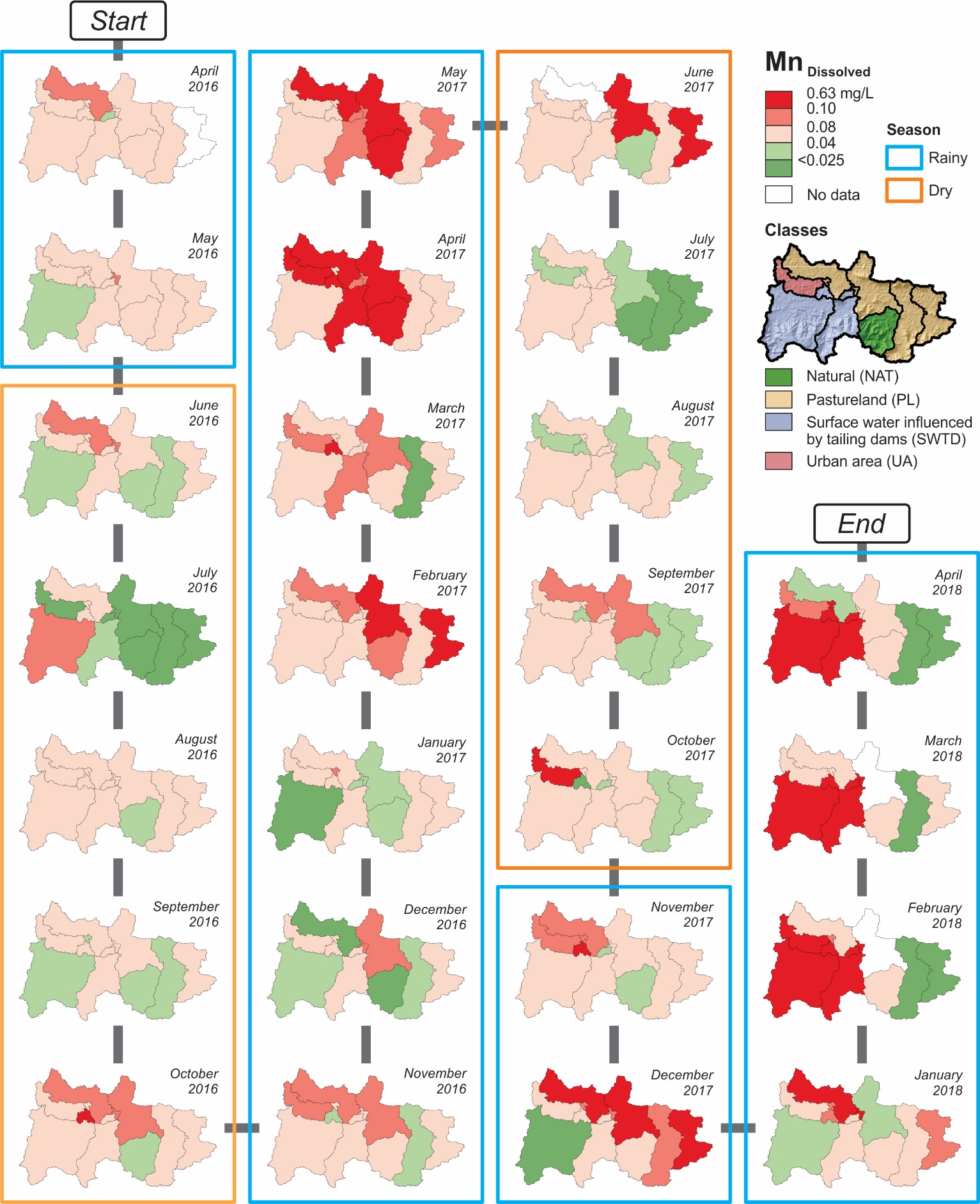


Appendix B.3. Time-series geochemical maps of Mn _Dissolved_ in surface water of the Gelado Creek Watershed.


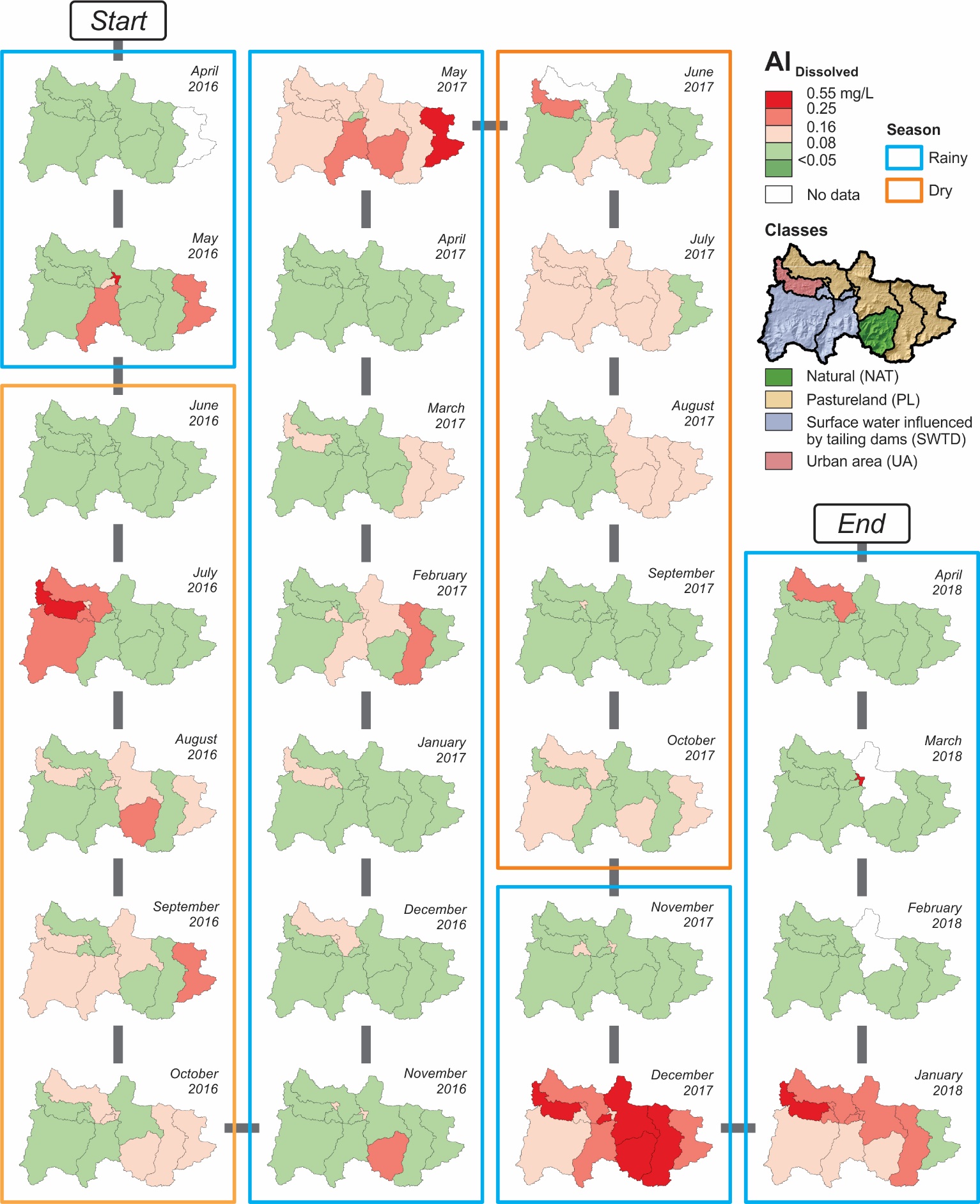


Appendix B.4. Time-series geochemical maps of Al _Dissolved_ in surface water of the Gelado Creek Watershed.


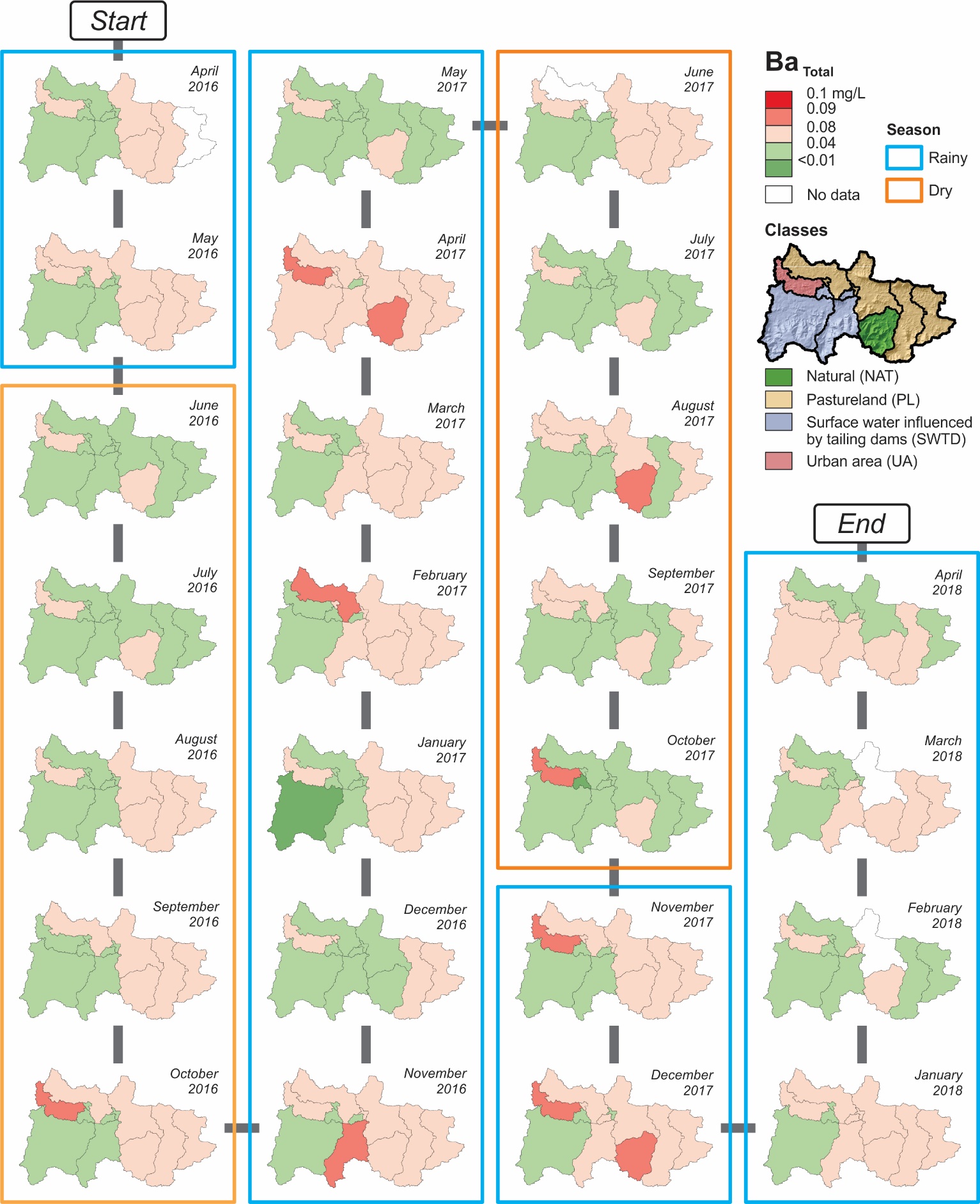


Appendix B.5. Time-series geochemical maps of Ba _Total_ in surface water of the Gelado Creek Watershed.
